# Supplementary material for: Association of overtime work hours with various stress responses in 59,021 Japanese workers: Retrospective cross-sectional study
Source: PLoS One. 2020 Mar 3;15(3):e0229506. doi: 10.1371/journal.pone.0229506 (PMC7053771; doi:10.1371/journal.pone.0229506)
Supplement: S1 Table — A Specific items with internal consistency of the scales of Brief Job Stress Questionnaire used in the study. B Correlation coefficient between stress response scales in BJSQ. (DOCX) [file pone.0229506.s001.docx]

| Supplemental table1-1: Specific items with internal consistency of the scales of Brief Job Stress Questionnaire used in the study | | | | | | |
| --- | --- | --- | --- | --- | --- | --- |
| Category | Scale | Number of item | Question | Mean | SD | Cronbach's alpha |
| Stressor |  |  | *Please answer the following questions concerning your job by circling the number that best fits your situation. (1:Very much so, 2: Moderately so, 3: Somewhat 4: Not at all)* | - | - | - |
|  | Job control | 3 | *I can work at my own pace* | 2.64 | 0.85 | 0.712 |
|  |  |  | *I can choose how and in what order to do my work* | 2.30 | 0.80 |  |
|  |  |  | *I can reflect my opinions on workplace policy* | 2.48 | 0.79 |  |
| Stress-response | |  | *Please answer the following questions concerning your health during the past month by circling the number that best fits your situation. (1:Almost never, 2: Sometimes 3: Often 4:Almost always)* | - | - | - |
|  | Vigor | 3 | *I have been very active* | 2.15 | 0.80 | 0.911 |
|  |  |  | *I have been full of energy* | 2.21 | 0.83 |  |
|  |  |  | *I have been lively* | 2.14 | 0.82 |  |
|  | Irritability | 3 | *I have felt angry* | 2.08 | 0.81 | 0.892 |
|  |  |  | *I have been inwardly annoyed or aggravated* | 2.06 | 0.84 |  |
|  |  |  | *I have felt irritable* | 2.10 | 0.82 |  |
|  | Fatigue | 3 | *I have felt extremely tired* | 2.40 | 0.86 | 0.873 |
|  |  |  | *I have felt exhausted* | 2.04 | 0.92 |  |
|  |  |  | *I have felt weary or listless* | 2.15 | 0.89 |  |
|  | Anxiety | 3 | *I have felt tense* | 2.34 | 0.88 | 0.764 |
|  |  |  | *I have felt worried or insecure* | 2.15 | 0.93 |  |
|  |  |  | *I have felt restless* | 1.80 | 0.84 |  |
|  | Depression | 6 | *I have been depressed* | 1.93 | 0.90 | 0.885 |
|  |  |  | *I have thought that doing anything was a hassle* | 1.76 | 0.81 |  |
|  |  |  | *I have been unable to concentrate* | 1.70 | 0.73 |  |
|  |  |  | *I have felt gloomy* | 1.96 | 0.85 |  |
|  |  |  | *I have been unable to handle work* | 1.42 | 0.64 |  |
|  |  |  | *I have felt sad* | 1.47 | 0.74 |  |
|  | Somatic responses | 11 | *I have felt dizzy* | 1.34 | 0.64 | 0.841 |
|  |  |  | *I have experienced joint pains* | 1.43 | 0.73 |  |
|  |  |  | *I have experienced headaches* | 1.70 | 0.81 |  |
|  |  |  | *I have had a stiff neck and / or shoulders* | 2.41 | 1.05 |  |
|  |  |  | *I have had lower back pain* | 1.94 | 0.96 |  |
|  |  |  | *I have had eyestrain* | 2.52 | 0.95 |  |
|  |  |  | *I have experienced heart palpitations or shortness of breath* | 1.32 | 0.63 |  |
|  |  |  | *I have experienced stomach and / or intestine problems* | 1.63 | 0.81 |  |
|  |  |  | *I have lost my appetite* | 1.31 | 0.60 |  |
|  |  |  | *I have experienced diarrhea and / or constipation* | 1.74 | 0.89 |  |
|  |  |  | *I haven’t been able to sleep well* | 1.66 | 0.84 |  |
| Support |  |  | *Please answer the following questions concerning people around you by circling the number that best fits your situation. (1: Extremely, 2:Very much, 3:Somewhat, 4:Not at all)* | - | - | - |
|  | Supervisor’s support | 3 | *How freely can you talk with your supervisor?* | 2.42 | 0.81 | 0.820 |
|  |  |  | *How reliable are your supervisor when you are troubled?* | 2.30 | 0.84 |  |
|  |  |  | *How well will your supervisor listen to you when you ask for advice on personal matters?* | 2.53 | 0.87 |  |
|  | Coworker’s support | 3 | *How freely can you talk with your coworker?* | 2.07 | 0.76 | 0.793 |
|  |  |  | *How reliable are your coworker when you are troubled?* | 2.26 | 0.78 |  |
|  |  |  | *How well will your coworker listen to you when you ask for advice on personal matters?* | 2.47 | 0.82 |  |

| Supplemental table 1-2: Correlation coefficient between stress response scales in BJSQ | | | | | | |
| --- | --- | --- | --- | --- | --- | --- |
|  | Lack of vigor | Irritability | Fatigue | Anxiety | Depression | Somatic responses |
| Lack of vigor | 1 |  |  |  |  |  |
| Irritability | 0.280*** | 1 |  |  |  |  |
| Fatigue | 0.341*** | 0.515*** | 1 |  |  |  |
| Anxiety | 0.281*** | 0.509*** | 0.623*** | 1 |  |  |
| Depression | 0.429*** | 0.553*** | 0.628*** | 0.704*** | 1 |  |
| Somatic responses | 0.298*** | 0.416*** | 0.581*** | 0.480*** | 0.583*** | 1 |

****p<0.001*

*Lack of vigor was derived by reversing the score of vigor for harmonization with other stress-response scales, which higher score indicates unfavorable stress-response*
