# Supplementary material for: Association of overtime work hours with various stress responses in 59,021 Japanese workers: Retrospective cross-sectional study
Source: PLoS One. 2020 Mar 3;15(3):e0229506. doi: 10.1371/journal.pone.0229506 (PMC7053771; doi:10.1371/journal.pone.0229506)
Supplement: S2 Table — (DOCX) [file pone.0229506.s002.docx]

| Supplemental table 2. Association between overtime work hours and stress responses : multiple linear regression results with multiple imputation | | | | | | | | | | | | | | | | | | | | | | | |
| --- | --- | --- | --- | --- | --- | --- | --- | --- | --- | --- | --- | --- | --- | --- | --- | --- | --- | --- | --- | --- | --- | --- | --- |
| Overtime work hours | Lack of Vigor | | |  | Irritability | | |  | Fatigue | | |  | Anxiety | | |  | Depression | | |  | Somatic responses | | |
|  | beta | 95％ CI | p |  | beta | 95％ CI | p |  | beta | 95％ CI | p |  | beta | 95％ CI | p |  | beta | 95％ CI | p |  | beta | 95％ CI | p |
| **Total** |  |  |  |  |  |  |  |  |  |  |  |  |  |  |  |  |  |  |  |  |  |  |  |
| ≤20 | Ref |  |  |  | Ref |  |  |  | Ref |  |  |  | Ref |  |  |  | Ref |  |  |  | Ref |  |  |
| 21-30 | 0.06 | (0.04 , 0.08) | *** |  | 0.14 | (0.12 , 0.16) | *** |  | 0.21 | (0.18 , 0.23) | *** |  | 0.15 | (0.13 , 0.17) | *** |  | 0.09 | (0.07 , 0.11) | *** |  | 0.07 | (0.04 , 0.09) | *** |
| 31-40 | 0.05 | (0.03 , 0.08) | *** |  | 0.19 | (0.16 , 0.21) | *** |  | 0.32 | (0.30 , 0.35) | *** |  | 0.23 | (0.20 , 0.25) | *** |  | 0.12 | (0.09 , 0.14) | *** |  | 0.12 | (0.09 , 0.14) | *** |
| 41-50 | 0.04 | (0.01 , 0.06) | ** |  | 0.21 | (0.18 , 0.24) | *** |  | 0.42 | (0.39 , 0.45) | *** |  | 0.28 | (0.25 , 0.31) | *** |  | 0.17 | (0.14 , 0.20) | *** |  | 0.17 | (0.14 , 0.19) | *** |
| 51-60 | -0.03 | (-0.06 , 0.00) | + |  | 0.24 | (0.20 , 0.27) | *** |  | 0.47 | (0.44 , 0.50) | *** |  | 0.32 | (0.29 , 0.35) | *** |  | 0.16 | (0.13 , 0.19) | *** |  | 0.17 | (0.14 , 0.20) | *** |
| 61-70 | -0.10 | (-0.14 , -0.06) | *** |  | 0.26 | (0.22 , 0.30) | *** |  | 0.54 | (0.50 , 0.58) | *** |  | 0.43 | (0.39 , 0.47) | *** |  | 0.22 | (0.18 , 0.26) | *** |  | 0.20 | (0.16 , 0.25) | *** |
| 71-80 | -0.12 | (-0.16 , -0.08) | *** |  | 0.35 | (0.31 , 0.40) | *** |  | 0.61 | (0.57 , 0.65) | *** |  | 0.50 | (0.46 , 0.55) | *** |  | 0.28 | (0.24 , 0.32) | *** |  | 0.27 | (0.23 , 0.31) | *** |
| 81- | 0.01 | (-0.05 , 0.07) |  |  | 0.40 | (0.34 , 0.40) | *** |  | 0.72 | (0.66 , 0.00) | *** |  | 0.46 | (0.40 , 0.51) | *** |  | 0.28 | (0.22 , 0.34) | *** |  | 0.25 | (0.19 , 0.31) | *** |
| p for linear trend | <0.001 (negative) | | |  | <0.001 (positive) | | |  | <0.001 (positive) | | |  | <0.001 (positive) | | |  | <0.001 (positive) | | |  | <0.001 (positive) | | |
| **Men** |  |  |  |  |  |  |  |  |  |  |  |  |  |  |  |  |  |  |  |  |  |  |  |
| ≤20 | Ref |  |  |  | Ref |  |  |  | Ref |  |  |  | Ref |  |  |  | Ref |  |  |  | Ref |  |  |
| 21-30 | 0.05 | (0.02 , 0.07) |  |  | 0.13 | (0.11 , 0.16) | *** |  | 0.16 | (0.14 , 0.19) | *** |  | 0.11 | (0.09 , 0.14) | *** |  | 0.05 | (0.02 , 0.07) | ** |  | 0.03 | (0.00 , 0.05) | * |
| 31-40 | 0.02 | (0.00 , 0.05) |  |  | 0.16 | (0.14 , 0.19) | *** |  | 0.27 | (0.24 , 0.29) | *** |  | 0.17 | (0.14 , 0.20) | *** |  | 0.07 | (0.04 , 0.09) | *** |  | 0.08 | (0.05 , 0.10) | *** |
| 41-50 | 0.01 | (-0.02 , 0.04) |  |  | 0.20 | (0.17 , 0.23) | *** |  | 0.37 | (0.34 , 0.40) | *** |  | 0.24 | (0.21 , 0.27) | *** |  | 0.13 | (0.10 , 0.16) | *** |  | 0.14 | (0.10 , 0.17) | *** |
| 51-60 | -0.03 | (-0.06 , 0.00) | + |  | 0.22 | (0.19 , 0.26) | *** |  | 0.43 | (0.39 , 0.46) | *** |  | 0.27 | (0.24 , 0.31) | *** |  | 0.11 | (0.08 , 0.14) | *** |  | 0.14 | (0.11 , 0.17) | *** |
| 61-70 | -0.12 | (-0.16 , -0.08) | *** |  | 0.25 | (0.20 , 0.29) | *** |  | 0.49 | (0.45 , 0.53) | *** |  | 0.38 | (0.34 , 0.42) | *** |  | 0.17 | (0.12 , 0.21) | *** |  | 0.16 | (0.11 , 0.20) | *** |
| 71-80 | -0.13 | (-0.17 , -0.08) | *** |  | 0.34 | (0.29 , 0.38) | *** |  | 0.57 | (0.52 , 0.61) | *** |  | 0.46 | (0.41 , 0.50) | *** |  | 0.23 | (0.18 , 0.27) | *** |  | 0.23 | (0.19 , 0.28) | *** |
| 81- | -0.01 | (-0.07 , 0.05) |  |  | 0.41 | (0.34 , 0.47) | *** |  | 0.72 | (0.66 , 0.78) | *** |  | 0.43 | (0.36 , 0.49) | *** |  | 0.25 | (0.19 , 0.31) | *** |  | 0.24 | (0.18 , 0.31) | *** |
| p for linear trend | <0.001 (negative) | | |  | <0.001 (positive) | | |  | <0.001 (positive) | | |  | <0.001 (positive) | | |  | <0.001 (positive) | | |  | <0.001 (positive) | | |
| **Women** |  |  |  |  |  |  |  |  |  |  |  |  |  |  |  |  |  |  |  |  |  |  |  |
| ≤20 | Ref |  |  |  | Ref |  |  |  | Ref |  |  |  | Ref |  |  |  | Ref |  |  |  | Ref |  |  |
| 21-30 | 0.08 | (0.04 , 0.12) | *** |  | 0.16 | (0.12 , 0.20) | *** |  | 0.28 | (0.24 , 0.31) | *** |  | 0.21 | (0.17 , 0.24) | *** |  | 0.16 | (0.12 , 0.19) | *** |  | 0.13 | (0.09 , 0.17) | *** |
| 31-40 | 0.15 | (0.10 , 0.20) | *** |  | 0.24 | (0.19 , 0.29) | *** |  | 0.46 | (0.41 , 0.51) | *** |  | 0.36 | (0.31 , 0.41) | *** |  | 0.21 | (0.16 , 0.26) | *** |  | 0.20 | (0.15 , 0.25) | *** |
| 41-50 | 0.14 | (0.07 , 0.21) | *** |  | 0.23 | (0.16 , 0.31) | *** |  | 0.53 | (0.46 , 0.61) | *** |  | 0.36 | (0.29 , 0.43) | *** |  | 0.24 | (0.16 , 0.31) | *** |  | 0.21 | (0.14 , 0.29) | *** |
| 51-60 | -0.03 | (-0.12 , 0.07) |  |  | 0.22 | (0.11 , 0.32) | *** |  | 0.59 | (0.49 , 0.69) | *** |  | 0.49 | (0.39 , 0.59) | *** |  | 0.34 | (0.24 , 0.43) | *** |  | 0.23 | (0.13 , 0.33) | *** |
| 61-70 | 0.11 | (-0.05 , 0.27) |  |  | 0.24 | (0.07 , 0.40) | ** |  | 0.77 | (0.61 , 0.93) | *** |  | 0.66 | (0.50 , 0.82) | *** |  | 0.54 | (0.38 , 0.69) | *** |  | 0.39 | (0.22 , 0.55) | *** |
| 71-80 | -0.11 | (-0.28 , 0.07) |  |  | 0.32 | (0.14 , 0.51) | ** |  | 0.78 | (0.60 , 0.97) | *** |  | 0.72 | (0.53 , 0.90) | *** |  | 0.60 | (0.42 , 0.77) | *** |  | 0.42 | (0.24 , 0.61) | *** |
| 81- | -0.03 | (-0.20 , 0.15) |  |  | 0.33 | (0.14 , 0.51) | *** |  | 0.57 | (0.39 , 0.76) | *** |  | 0.51 | (0.33 , 0.69) | *** |  | 0.35 | (0.17 , 0.52) | ** |  | 0.20 | (0.01 , 0.38) | * |
| p for linear trend | <0.001 (positive) | | |  | <0.001 (positive) | | |  | <0.001 (positive) | | |  | <0.001 (positive) | | |  | <0.001 (positive) | | |  | <0.001 (positive) | | |

+ p<0.1, *p<0.05, ** p<0.01, ***p<0.001, Higher score indicates unfavorable stress-response. All betas were adjusted by age, type of job, job class, employment status, type of schedule, company size, company industry, job control, supervisors support and coworker’s support. Lack of vigor was derived by reversing the score of vigor for harmonization with other stress-response scales, which higher score indicates unfavorable stress-response
