# Supplementary material for: Association of overtime work hours with various stress responses in 59,021 Japanese workers: Retrospective cross-sectional study
Source: PLoS One. 2020 Mar 3;15(3):e0229506. doi: 10.1371/journal.pone.0229506 (PMC7053771; doi:10.1371/journal.pone.0229506)
Supplement: Attachment — Submitted filename: [Plos One, R1] Response letter ver5.0 for reviewes.docx [file pone.0229506.s003.docx]

Reviewer #1

First, we appreciate your positive evaluation of the present study and useful comments on our manuscript. As Reviewer #2 pointed out, we changed the title of this manuscript into **“Association of overtime work hours with various stress responses in 59,021 Japanese workers: retrospective cross-sectional study”.**

***Comment #1***

1. **Theory**

**As far as I have understood the PLOSone does not emphasizes *theoretical* contributions to the literature. However, there are a couple of meta-analyses and large survey studies about overtime. Hence, stating clearly how this study adds to the literature would be beneficial. You argue that your sample is stratified and the others were not, but why is that important?**

(Our response for these comments)

We thank you for this important comment. We agree to clarify how this study adds new evidence over existing research. As stated in the manuscript, past studies have not sufficiently focused on gender-stratified analysis due to limited number of women working long hours. Considering the growing number of working women world-wide, evidence regarding working women becomes more and more important. In addition, we received the suggestion from Reviewer 2 to conduct age-stratified analysis. Since the number of older workers will be increasing in this ageing society, age-stratified analysis would also be important.

This study is based upon a large sample, which enables us to conduct age and gender stratified analyses. Therefore, in response to the reviewers’ comments, we added several stratified analyses, together with analysis of effect modification by gender, age and job-position. These additional analyses are needed for clarification of an accurate picture of the association between overtime work and stress responses.

(Introduction section, page 5-6, line 68-78)

*In this context, it is important to consider the upper overtime-limit to prevent mental illness among workers. So far, several studies have investigated associations between long working hours and mental health (1,14). However, many studies used tertile (15,16), quartile (17–19) or quintile (20,21) cut-off were used for evaluating effects of amount of overtime work. Studies in which a broader range of overtime is used are needed for the establishment of an upper overtime-limit as a health policy. In addition, there are only few published in which gender-stratified analysis has been performed. This is due to the limited number of women with long working hours (19,22). Labor force participation among women of working age increased substantially worldwide (23). Furthermore, occupational class might influence the relation between overtime and health outcome (24). To nuance the picture of associations between overtime and mental health, stratified analysis by these factors is also needed.*

(Materials and methods section, page 11, line 168-171)

*Finally, stratified analyses by gender, age-category (i.e. less than 30, 30-59 and 60 or older) and job-position (regular and manager) were also performed. In these models, interaction terms between age-category/job-position and overtime working hours were used to explore possible effect modification.*

(Results and discussion section, page 17-18, line 234-246)

*For gender differences, significant interactions by gender were observed in the association between overtime working hours and several stress responses. Compared to workers with shorter overtime working hours, those with longer hours tend to have higher stress responses for both genders; however, this association was stronger among women than men. This implies that working women may be more vulnerable than men for long working hours.*

*Table 3 and 4 showed stratified analysis results by age-category and job-class. Compared to middle-aged male workers, older workers showed relatively higher stress responses, especially fatigue, anxiety and depression. This may suggest that older workers constitute a more vulnerable population than younger ones for overtime working than middle-aged workers. Due to the small number of working women in higher job-positions, the interaction by job-class was not clear. It is interesting that the highest beta coefficient in point estimates was observed for fatigue among women in administrative positions (beta=1.16 [0.68. 1.63]).*

(Added tables)

Table 3: Association between overtime work hours and stress responses: results of multiple linear regression stratified by gender and age-category

Table 4. Association between overtime work hours and stress responses; results of multiple linear regression stratified by gender and job-class

| **Table 2**. Associations between overtime work hours and stress responses: gender-stratified multiple linear regression results | | | | | | | | | | | | | | | | | | | | | | | | |
| --- | --- | --- | --- | --- | --- | --- | --- | --- | --- | --- | --- | --- | --- | --- | --- | --- | --- | --- | --- | --- | --- | --- | --- | --- |
| Overtime working hours/month | n | Lack of Vigor | | |  | Irritability | | |  | Fatigue | | |  | Anxiety | | |  | Depression | | |  | Somatic responses | | |
|  |  | beta | 95％ CI | p |  | beta | 95％ CI | p |  | beta | 95％ CI | p |  | beta | 95％ CI | p |  | beta | 95％ CI | p |  | beta | 95％ CI | p |
| **Men** |  |  |  |  |  |  |  |  |  |  |  |  |  |  |  |  |  |  |  |  |  |  |  |  |
| ≤20 | 15,751 | Ref |  |  |  | Ref |  |  |  | Ref |  |  |  | Ref |  |  |  | Ref |  |  |  | Ref |  |  |
| 21-30 | 7,260 | 0.04 | (0.02 , 0.07) | ** |  | 0.13 | (0.10 , 0.16) | *** |  | 0.16 | (0.13 , 0.18) | *** |  | 0.11 | (0.08 , 0.14) | *** |  | 0.04 | (0.02 , 0.07) | ** |  | 0.03 | (0.00 , 0.05) | * |
| 31-40 | 5,812 | 0.01 | (-0.02 , 0.04) |  |  | 0.17 | (0.14 , 0.19) | *** |  | 0.26 | (0.23 , 0.29) | *** |  | 0.16 | (0.13 , 0.19) | *** |  | 0.06 | (0.03 , 0.08) | *** |  | 0.07 | (0.04 , 0.10) | *** |
| 41-50 | 4,097 | 0.00 | (-0.03 , 0.03) |  |  | 0.19 | (0.16 , 0.22) | *** |  | 0.36 | (0.33 , 0.39) | *** |  | 0.23 | (0.20 , 0.26) | *** |  | 0.12 | (0.09 , 0.15) | *** |  | 0.13 | (0.10 , 0.17) | *** |
| 51-60 | 3,494 | -0.04 | (-0.07 , 0.00) | * |  | 0.22 | (0.18 , 0.25) | *** |  | 0.42 | (0.38 , 0.45) | *** |  | 0.26 | (0.23 , 0.29) | *** |  | 0.10 | (0.07 , 0.13) | *** |  | 0.14 | (0.10 , 0.17) | *** |
| 61-70 | 1,814 | -0.13 | (-0.17 , -0.09) | *** |  | 0.23 | (0.19 , 0.28) | *** |  | 0.48 | (0.44 , 0.53) | *** |  | 0.38 | (0.33 , 0.42) | *** |  | 0.16 | (0.12 , 0.21) | *** |  | 0.16 | (0.11 , 0.21) | *** |
| 71-80 | 1,740 | -0.13 | (-0.18 , -0.09) | *** |  | 0.33 | (0.28 , 0.38) | *** |  | 0.57 | (0.52 , 0.61) | *** |  | 0.44 | (0.40 , 0.49) | *** |  | 0.22 | (0.17 , 0.26) | *** |  | 0.23 | (0.18 , 0.27) | *** |
| 81- | 796 | -0.01 | (-0.07 , 0.06) |  |  | 0.41 | (0.35 , 0.48) | *** |  | 0.73 | (0.66 , 0.79) | *** |  | 0.42 | (0.35 , 0.48) | *** |  | 0.24 | (0.17 , 0.30) | *** |  | 0.23 | (0.16 , 0.29) | *** |
| p for linear trend | | <0.001 (negative) | | |  | <0.001 (positive) | | |  | <0.001 (positive) | | |  | <0.001 (positive) | | |  | <0.001 (positive) | | |  | <0.001 (positive) | | |
| **Women** |  |  |  |  |  |  |  |  |  |  |  |  |  |  |  |  |  |  |  |  |  |  |  |  |
| ≤20 | 13,529 | Ref |  |  |  | Ref |  |  |  | Ref |  |  |  | Ref |  |  |  | Ref |  |  |  | Ref |  |  |
| 21-30 | 2,301 | 0.07 | (0.03 , 0.11) | *** |  | 0.15 | (0.10 , 0.19) | *** |  | 0.26 | (0.21 , 0.30) | *** |  | 0.19 | (0.14 , 0.23) | *** |  | 0.13 | (0.09 , 0.17) | *** |  | 0.11 | (0.07 , 0.16) | *** |
| 31-40 | 1,219 | 0.15 | (0.10 , 0.20) | *** |  | 0.23 | (0.17 , 0.28) | *** |  | 0.47 | (0.21 , 0.31) | *** |  | 0.36 | (0.31 , 0.42) | *** |  | 0.20 | (0.15 , 0.25) | *** |  | 0.19 | (0.13 , 0.25) | *** |
| 41-50 | 567 | 0.14 | (0.06 , 0.22) | *** |  | 0.20 | (0.12 , 0.29) | *** |  | 0.52 | (0.21 , 0.32) | *** |  | 0.34 | (0.26 , 0.42) | *** |  | 0.22 | (0.14 , 0.30) | *** |  | 0.18 | (0.10 , 0.26) | *** |
| 51-60 | 326 | -0.04 | (-0.14 , 0.07) |  |  | 0.21 | (0.11 , 0.32) | *** |  | 0.60 | (0.21 , 0.33) | *** |  | 0.50 | (0.39 , 0.61) | *** |  | 0.33 | (0.23 , 0.43) | *** |  | 0.25 | (0.14 , 0.36) | *** |
| 61-70 | 127 | 0.12 | (-0.04 , 0.28) |  |  | 0.25 | (0.08 , 0.42) | ** |  | 0.82 | (0.21 , 0.34) | *** |  | 0.71 | (0.54 , 0.88) | *** |  | 0.54 | (0.38 , 0.70) | *** |  | 0.41 | (0.24 , 0.58) | *** |
| 71-80 | 97 | -0.14 | (-0.32 , 0.05) |  |  | 0.39 | (0.19 , 0.58) | *** |  | 0.78 | (0.21 , 0.35) | *** |  | 0.71 | (0.52 , 0.90) | *** |  | 0.60 | (0.42 , 0.79) | *** |  | 0.44 | (0.24 , 0.63) | *** |
| 81- | 91 | 0.01 | (-0.18 , 0.20) |  |  | 0.32 | (0.12 , 0.52) | ** |  | 0.62 | (0.21 , 0.36) | *** |  | 0.53 | (0.33 , 0.72) | *** |  | 0.34 | (0.15 , 0.52) | ** |  | 0.13 | (-0.07 , 0.33) |  |
| p for linear trend | | <0.001 (positive) | | |  | <0.001 (positive) | | |  | <0.001 (positive) | | |  | <0.001 (positive) | | |  | <0.001 (positive) | | |  | <0.001 (positive) | | |
| **Interaction terms** | |  |  |  |  |  |  |  |  |  |  |  |  |  |  |  |  |  |  |  |  |  |  |  |
| **Men (ref) versus Women** | | |  |  |  |  |  |  |  |  |  |  |  |  |  |  |  |  |  |  |  |  |  |  |
| **21-30** |  | **0.04** | **(-0.01 , 0.08)** |  |  | **0.02** | **(-0.03 , 0.06)** |  |  | **0.09** | **(0.04 , 0.13)** | ******* |  | **0.07** | **(0.02 , 0.11)** | ****** |  | **0.08** | **(0.03 , 0.13)** | ****** |  | **0.09** | **(0.04 , 0.14)** | ******* |
| **31-40** |  | **0.15** | **(0.09 , 0.21)** | ******* |  | **0.06** | **(0.00 , 0.12)** | **+** |  | **0.19** | **(0.13 , 0.25)** | ******* |  | **0.18** | **(0.12 , 0.24)** | ******* |  | **0.13** | **(0.07 , 0.19)** | ******* |  | **0.12** | **(0.06 , 0.19)** | ******* |
| **41-50** |  | **0.15** | **(0.06 , 0.23)** | ******* |  | **0.01** | **(-0.08 , 0.09)** |  |  | **0.13** | **(0.05 , 0.22)** | ****** |  | **0.08** | **(0.00 , 0.16)** | **+** |  | **0.08** | **(0.00 , 0.17)** | ***** |  | **0.04** | **(-0.04 , 0.13)** |  |
| **51-60** |  | **0.04** | **(-0.06 , 0.14)** |  |  | **-0.03** | **(-0.14 , 0.08)** |  |  | **0.13** | **(0.03 , 0.24)** | ***** |  | **0.18** | **(0.08 , 0.29)** | ****** |  | **0.20** | **(0.09 , 0.30)** | ******* |  | **0.09** | **(-0.02 , 0.20)** |  |
| **61-70** |  | **0.29** | **(0.12 , 0.45)** | ****** |  | **-0.02** | **(-0.19 , 0.15)** |  |  | **0.30** | **(0.13 , 0.46)** | ******* |  | **0.28** | **(0.12 , 0.45)** | ****** |  | **0.35** | **(0.18 , 0.51)** | ******* |  | **0.21** | **(0.04 , 0.38)** | ***** |
| **71-80** |  | **0.03** | **(-0.15 , 0.22)** |  |  | **0.01** | **(-0.18 , 0.21)** |  |  | **0.16** | **(-0.03 , 0.35)** | **+** |  | **0.20** | **(0.01 , 0.39)** | ***** |  | **0.34** | **(0.16 , 0.53)** | ******* |  | **0.17** | **(-0.03 , 0.36)** | **+** |
| **81-** |  | **-0.01** | **(-0.20 , 0.19)** |  |  | **-0.09** | **(-0.29 , 0.12)** |  |  | **-0.09** | **(-0.29 , 0.11)** |  |  | **0.10** | **(-0.10 , 0.30)** |  |  | **0.11** | **(-0.09 , 0.31)** |  |  | **-0.08** | **(-0.29 , 0.12)** |  |

+ p<0.1, *p<0.05, ** p<0.01, ***p<0.001, Higher score indicates unfavorable stress-response. All betas were adjusted by age, type of job, job class, employment status, type of schedule, company size, company industry, job control, supervisors support and coworker’s support. Lack of vigor was derived by reversing the score of vigor for harmonization with other stress-response scales, which higher score indicates unfavorable stress-response

| **Table 3 . Association between overtime work hours and stress responses: results of multiple linear regression stratified by gender and age-category** | | | | | | | | | | | | | | | | | | | | | | | | |
| --- | --- | --- | --- | --- | --- | --- | --- | --- | --- | --- | --- | --- | --- | --- | --- | --- | --- | --- | --- | --- | --- | --- | --- | --- |
| **Overtime work**  **hours/month** | **n** | **Lack of Vigor** | | |  | **Irritability** | | |  | **Fatigue** | | |  | **Anxiety** | | |  | **Depression** | | |  | **Somatic responses** | | |
|  |  | **beta** | **95％ CI** | **p** |  | **beta** | **95％ CI** | **p** |  | **beta** | **95％ CI** | **p** |  | **beta** | **95％ CI** | **p** |  | **beta** | **95％ CI** | **p** |  | **beta** | **95％ CI** | **p** |
| **Men** |  |  |  |  |  |  |  |  |  |  |  |  |  |  |  |  |  |  |  |  |  |  |  |  |
| **Younger (aged less than 30 years)** | | | |  |  |  |  |  |  |  |  |  |  |  |  |  |  |  |  |  |  |  |  |  |
| **≤20** | **1,485** | **Ref** |  |  |  | **Ref** |  |  |  | **Ref** |  |  |  | **Ref** |  |  |  | **Ref** |  |  |  | **Ref** |  |  |
| **21-30** | **796** | **0.12** | **(0.04 , 0.20)** | ****** |  | **0.15** | **(0.07 , 0.24)** | ******* |  | **0.22** | **(0.14 , 0.30)** | ******* |  | **0.12** | **(0.03 , 0.20)** | ****** |  | **0.12** | **(0.04 , 0.21)** | ****** |  | **0.03** | **(-0.06 , 0.12)** |  |
| **31-40** | **546** | **0.08** | **(-0.01 , 0.17)** | **+** |  | **0.17** | **(0.07 , 0.27)** | ****** |  | **0.28** | **(0.19 , 0.37)** | ******* |  | **0.15** | **(0.05 , 0.24)** | ****** |  | **0.06** | **(-0.03 , 0.16)** |  |  | **0.10** | **(0.00 , 0.20)** |  |
| **41-50** | **345** | **0.08** | **(-0.03 , 0.18)** |  |  | **0.21** | **(0.09 , 0.33)** | ****** |  | **0.45** | **(0.35 , 0.56)** | ******* |  | **0.30** | **(0.19 , 0.42)** | ******* |  | **0.28** | **(0.17 , 0.40)** | ******* |  | **0.32** | **(0.20 , 0.44)** | ******* |
| **51-60** | **291** | **-0.05** | **(-0.17 , 0.07)** |  |  | **0.29** | **(0.16 , 0.42)** | ******* |  | **0.48** | **(0.36 , 0.59)** | ******* |  | **0.28** | **(0.15 , 0.41)** | ******* |  | **0.14** | **(0.01 , 0.26)** | ***** |  | **0.15** | **(0.01 , 0.28)** | ***** |
| **61-70** | **146** | **-0.01** | **(-0.16 , 0.14)** |  |  | **0.35** | **(0.18 , 0.52)** | ******* |  | **0.57** | **(0.42 , 0.72)** | ******* |  | **0.49** | **(0.33 , 0.66)** | ******* |  | **0.37** | **(0.20 , 0.54)** | ****** |  | **0.30** | **(0.13 , 0.48)** | ******* |
| **71-80** | **135** | **-0.12** | **(-0.28 , 0.04)** |  |  | **0.40** | **(0.22 , 0.58)** | ******* |  | **0.42** | **(0.26 , 0.58)** | ******* |  | **0.51** | **(0.34 , 0.69)** | ******* |  | **0.41** | **(0.23 , 0.59)** | ******* |  | **0.38** | **(0.20 , 0.56)** | ******* |
| **81-** | **77** | **0.05** | **(-0.16 , 0.25)** |  |  | **0.48** | **(0.26 , 0.71)** | ******* |  | **0.89** | **(0.69 , 1.09)** | ******* |  | **0.44** | **(0.22 , 0.66)** | ******* |  | **0.44** | **(0.22 , 0.66)** | ****** |  | **0.27** | **(0.04 , 0.50)** | ***** |
| **p for linear trend** | | **<0.001 (negative)** | | |  | **<0.001 (positive)** | | |  | **<0.001 (positive)** | | |  | **<0.001 (positive)** | | |  | **<0.001 (positive)** | | |  | **<0.001 (positive)** | | |
| **Middle (aged 30-59 years)** | | |  |  |  |  |  |  |  |  |  |  |  |  |  |  |  |  |  |  |  |  |  |  |
| **≤20** | **12,097** | **Ref** |  |  |  | **Ref** |  |  |  | **Ref** |  |  |  | **Ref** |  |  |  | **Ref** |  |  |  | **Ref** |  |  |
| **21-30** | **6,132** | **0.01** | **(-0.02 , 0.03)** |  |  | **0.11** | **(0.08 , 0.14)** | ******* |  | **0.13** | **(0.10 , 0.16)** | ******* |  | **0.08** | **(0.05 , 0.11)** | ******* |  | **0.01** | **(-0.02 , 0.04)** |  |  | **0.01** | **(-0.02 , 0.04)** |  |
| **31-40** | **5,038** | **-0.03** | **(-0.06 , 0.00)** | ***** |  | **0.14** | **(0.11 , 0.17)** | ******* |  | **0.24** | **(0.21 , 0.27)** | ******* |  | **0.13** | **(0.10 , 0.16)** | ******* |  | **0.03** | **(0.00 , 0.06)** | **+** |  | **0.04** | **(0.01 , 0.07)** | ****** |
| **41-50** | **3,624** | **-0.04** | **(-0.07 , 0.00)** | ***** |  | **0.16** | **(0.13 , 0.20)** | ******* |  | **0.33** | **(0.30 , 0.36)** | ******* |  | **0.19** | **(0.15 , 0.22)** | ******* |  | **0.08** | **(0.04 , 0.11)** | ******* |  | **0.09** | **(0.06 , 0.13)** | ******* |
| **51-60** | **3,129** | **-0.07** | **(-0.10 , -0.03)** | ******* |  | **0.19** | **(0.15 , 0.22)** | ******* |  | **0.39** | **(0.35 , 0.42)** | ******* |  | **0.22** | **(0.19 , 0.26)** | ******* |  | **0.07** | **(0.04 , 0.11)** | ******* |  | **0.11** | **(0.08 , 0.15)** | ******* |
| **61-70** | **1,617** | **-0.17** | **(-0.21 , -0.12)** | ******* |  | **0.20** | **(0.16 , 0.25)** | ******* |  | **0.45** | **(0.41 , 0.50)** | ******* |  | **0.33** | **(0.28 , 0.37)** | ******* |  | **0.12** | **(0.07 , 0.17)** | ******* |  | **0.13** | **(0.08 , 0.18)** | ******* |
| **71-80** | **1,577** | **-0.17** | **(-0.22 , -0.12)** | ******* |  | **0.30** | **(0.25 , 0.35)** | ******* |  | **0.56** | **(0.51 , 0.61)** | ******* |  | **0.40** | **(0.35 , 0.45)** | ******* |  | **0.18** | **(0.13 , 0.22)** | ******* |  | **0.19** | **(0.14 , 0.24)** | ******* |
| **81-** | **683** | **-0.04** | **(-0.10 , 0.03)** |  |  | **0.40** | **(0.33 , 0.47)** | ******* |  | **0.70** | **(0.63 , 0.77)** | ******* |  | **0.40** | **(0.33 , 0.47)** | ******* |  | **0.20** | **(0.13 , 0.27)** | ******* |  | **0.20** | **(0.13 , 0.27)** | ******* |
| **p for linear trend** | | **<0.001 (negative)** | | |  | **<0.001 (positive)** | | |  | **<0.001 (positive)** | | |  | **<0.001 (positive)** | | |  | **<0.001 (positive)** | | |  | **<0.001 (positive)** | | |
| **Older (aged 60 or older)** | | |  |  |  |  |  |  |  |  |  |  |  |  |  |  |  |  |  |  |  |  |  |  |
| **≤20** | **2,169** | **Ref** |  |  |  | **Ref** |  |  |  | **Ref** |  |  |  | **Ref** |  |  |  | **Ref** |  |  |  | **Ref** |  |  |
| **21-30** | **332** | **0.01** | **(-0.09 , 0.12)** |  |  | **0.08** | **(-0.02 , 0.18)** |  |  | **0.24** | **(0.15 , 0.33)** | ******* |  | **0.23** | **(0.13 , 0.32)** | ******* |  | **0.13** | **(0.03 , 0.22)** | ****** |  | **0.07** | **(-0.04 , 0.17)** |  |
| **31-40** | **228** | **0.09** | **(-0.03 , 0.22)** |  |  | **0.29** | **(0.17 , 0.41)** | ******* |  | **0.34** | **(0.23 , 0.45)** | ******* |  | **0.31** | **(0.20 , 0.42)** | ******* |  | **0.23** | **(0.12 , 0.34)** | ******* |  | **0.22** | **(0.09 , 0.34)** | ****** |
| **41-50** | **128** | **-0.06** | **(-0.22 , 0.10)** |  |  | **0.26** | **(0.10 , 0.41)** | ****** |  | **0.52** | **(0.37 , 0.66)** | ******* |  | **0.43** | **(0.28 , 0.57)** | ******* |  | **0.30** | **(0.16 , 0.45)** | ******* |  | **0.30** | **(0.13 , 0.46)** | ******* |
| **51-60** | **74** | **-0.04** | **(-0.25 , 0.17)** |  |  | **0.27** | **(0.06 , 0.46)** | ***** |  | **0.57** | **(0.38 , 0.75)** | ******* |  | **0.48** | **(0.29 , 0.66)** | ******* |  | **0.30** | **(0.11 , 0.48)** | ****** |  | **0.37** | **(0.16 , 0.58)** | ****** |
| **61-70** | **51** | **-0.36** | **(-0.61 , -0.11)** | ****** |  | **0.15** | **(-0.09 , 0.39)** |  |  | **0.50** | **(0.28 , 0.72)** | ******* |  | **0.51** | **(0.29 , 0.73)** | ******* |  | **0.20** | **(-0.02 , 0.42)** | **+** |  | **0.20** | **(-0.05 , 0.45)** |  |
| **71-80** | **28** | **0.14** | **(-0.20 , 0.47)** |  |  | **0.40** | **(0.08 , 0.72)** | ***** |  | **0.68** | **(0.38 , 0.97)** | ******* |  | **0.73** | **(0.44 , 1.03)** | ******* |  | **0.31** | **(0.02 , 0.61)** | ***** |  | **0.47** | **(0.14 , 0.81)** | ****** |
| **81-** | **36** | **0.05** | **(-0.25 , 0.35)** |  |  | **0.10** | **(-0.18 , 0.38)** |  |  | **0.59** | **(0.33 , 0.85)** | ******* |  | **0.32** | **(0.06 , 0.58)** | ****** |  | **0.16** | **(-0.11 , 0.42)** |  |  | **0.29** | **(-0.01 , 0.58)** | **+** |
| **p for linear trend** | | **n.s.** | | |  | **<0.001 (positive)** | | |  | **<0.001 (positive)** | | |  | **<0.001 (positive)** | | |  | **<0.001 (positive)** | | |  | **<0.001 (positive)** | | |
| **Interaction between overtime and age-category** | | | | |  |  |  |  |  |  |  |  |  |  |  |  |  |  |  |  |  |  |  |  |
| **Middle (ref) versus Young** | | |  |  |  |  |  |  |  |  |  |  |  |  |  |  |  |  |  |  |  |  |  |  |
| **21-30** |  | **0.14** | **(0.05 , 0.21)** | ****** |  | **0.04** | **(-0.04 , 0.13)** |  |  | **0.06** | **(-0.02 , 0.15)** |  |  | **0.03** | **(-0.05 , 0.11)** |  |  | **0.11** | **(0.03 , 0.19)** | ***** |  | **0.04** | **(-0.05 , 0.13)** |  |
| **31-40** |  | **0.16** | **(0.07 , 0.25)** | ****** |  | **0.04** | **(-0.06 , 0.13)** |  |  | **0.03** | **(-0.07 , 0.12)** |  |  | **0.01** | **(-0.08 , 0.10)** |  |  | **0.04** | **(-0.06 , 0.13)** |  |  | **0.08** | **(-0.02 , 0.17)** |  |
| **41-50** |  | **0.16** | **(0.05 , 0.27)** | ****** |  | **0.06** | **(-0.05 , 0.17)** |  |  | **0.10** | **(-0.01 , 0.21)** |  |  | **0.10** | **(-0.02 , 0.21)** |  |  | **0.21** | **(0.10 , 0.32)** | ******* |  | **0.24** | **(0.13 , 0.36)** | ******* |
| **51-60** |  | **0.09** | **(-0.02 , 0.21)** |  |  | **0.12** | **(0.00 , 0.24)** | ***** |  | **0.07** | **(-0.05 , 0.18)** |  |  | **0.04** | **(-0.08 , 0.15)** |  |  | **0.08** | **(-0.04 , 0.20)** |  |  | **0.06** | **(-0.06 , 0.18)** |  |
| **61-70** |  | **0.24** | **(0.08 , 0.39)** | ****** |  | **0.18** | **(0.01 , 0.34)** | ***** |  | **0.13** | **(-0.03 , 0.29)** |  |  | **0.17** | **(0.01 , 0.33)** | ***** |  | **0.30** | **(0.14 , 0.46)** | ******* |  | **0.20** | **(0.03 , 0.37)** | ***** |
| **71-80** |  | **0.13** | **(-0.03 , 0.29)** |  |  | **0.12** | **(-0.05 , 0.28)** |  |  | **-0.14** | **(-0.30 , 0.02)** | **+** |  | **0.11** | **(-0.06 , 0.27)** |  |  | **0.27** | **(0.10 , 0.44)** | ****** |  | **0.22** | **(0.05 , 0.39)** | ***** |
| **81-** |  | **0.08** | **(-0.13 , 0.29)** |  |  | **0.08** | **(-0.14 , 0.30)** |  |  | **0.14** | **(-0.07 , 0.36)** |  |  | **0.01** | **(-0.21 , 0.22)** |  |  | **0.19** | **(-0.03 , 0.41)** | **+** |  | **0.06** | **(-0.16 , 0.29)** |  |
| **Middle (ref) versus Older** | | |  |  |  |  |  |  |  |  |  |  |  |  |  |  |  |  |  |  |  |  |  |  |
| **21-30** |  | **0.06** | **(-0.04 , 0.17)** |  |  | **-0.01** | **(-0.12 , 0.10)** |  |  | **0.12** | **(0.02 , 0.23)** | ***** |  | **0.16** | **(0.05 , 0.27)** | ****** |  | **0.14** | **(0.03 , 0.24)** | ***** |  | **0.10** | **(-0.01 , 0.21)** | **+** |
| **31-40** |  | **0.21** | **(0.09 , 0.33)** | ****** |  | **0.20** | **(0.07 , 0.33)** | ***** |  | **0.14** | **(0.01 , 0.26)** | ***** |  | **0.19** | **(0.07 , 0.32)** | ****** |  | **0.22** | **(0.10 , 0.35)** | ****** |  | **0.23** | **(0.10 , 0.36)** | ******* |
| **41-50** |  | **0.09** | **(-0.07 , 0.25)** |  |  | **0.17** | **(0.00 , 0.33)** | **+** |  | **0.25** | **(0.09 , 0.41)** | ***** |  | **0.25** | **(0.09 , 0.42)** | ****** |  | **0.27** | **(0.10 , 0.43)** | ****** |  | **0.27** | **(0.10 , 0.44)** | ****** |
| **51-60** |  | **0.15** | **(-0.05 , 0.36)** |  |  | **0.16** | **(-0.05 , 0.38)** |  |  | **0.24** | **(0.03 , 0.45)** | ***** |  | **0.25** | **(0.04 , 0.46)** | ***** |  | **0.27** | **(0.05 , 0.48)** | ***** |  | **0.33** | **(0.11 , 0.55)** | ****** |
| **61-70** |  | **-0.08** | **(-0.33 , 0.17)** |  |  | **0.04** | **(-0.22 , 0.29)** |  |  | **0.12** | **(-0.13 , 0.38)** |  |  | **0.20** | **(-0.06 , 0.45)** |  |  | **0.14** | **(-0.12 , 0.40)** |  |  | **0.14** | **(-0.12 , 0.41)** |  |
| **71-80** |  | **0.40** | **(0.07 , 0.73)** | ***** |  | **0.18** | **(-0.16 , 0.53)** |  |  | **0.18** | **(-0.15 , 0.51)** |  |  | **0.35** | **(0.01 , 0.69)** | ***** |  | **0.21** | **(-0.13 , 0.55)** |  |  | **0.36** | **(0.01 , 0.72)** | ***** |
| **81-** |  | **0.09** | **(-0.20 , 0.39)** |  |  | **-0.30** | **(-0.61 , 0.01)** | **+** |  | **-0.15** | **(-0.45 , 0.16)** |  |  | **-0.06** | **(-0.36 , 0.25)** |  |  | **-0.02** | **(-0.32 , 0.29)** |  |  | **0.08** | **(-0.23 , 0.40)** |  |
| **Women** |  |  |  |  |  |  |  |  |  |  |  |  |  |  |  |  |  |  |  |  |  |  |  |  |
| **Younger (aged less than 30 years)** | | | |  |  |  |  |  |  |  |  |  |  |  |  |  |  |  |  |  |  |  |  |  |
| **≤20** | **1,665** | **Ref** |  |  |  | **Ref** |  |  |  | **Ref** |  |  |  | **Ref** |  |  |  | **Ref** |  |  |  | **Ref** |  |  |
| **21-30** | **473** | **0.02** | **(-0.07 , 0.11)** |  |  | **0.13** | **(0.02 , 0.23)** | ***** |  | **0.23** | **(0.14 , 0.33)** | ******* |  | **0.09** | **(-0.02 , 0.19)** | **+** |  | **0.10** | **(0.00 , 0.20)** | ***** |  | **0.08** | **(-0.02 , 0.18)** |  |
| **31-40** | **246** | **0.14** | **(0.01 , 0.26)** | ***** |  | **0.18** | **(0.04 , 0.32)** | ***** |  | **0.37** | **(0.24 , 0.49)** | ******* |  | **0.25** | **(0.12 , 0.38)** | ******* |  | **0.26** | **(0.13 , 0.39)** | ******* |  | **0.06** | **(-0.08 , 0.20)** |  |
| **41-50** | **96** | **0.11** | **(-0.08 , 0.29)** |  |  | **0.10** | **(-0.11 , 0.31)** |  |  | **0.46** | **(0.26 , 0.65)** | ******* |  | **0.34** | **(0.13 , 0.54)** | ****** |  | **0.23** | **(0.03 , 0.43)** | ***** |  | **0.09** | **(-0.12 , 0.30)** |  |
| **51-60** | **81** | **0.11** | **(-0.09 , 0.32)** |  |  | **0.11** | **(-0.12 , 0.34)** |  |  | **0.66** | **(0.44 , 0.87)** | ******* |  | **0.47** | **(0.25 , 0.69)** | ******* |  | **0.47** | **(0.25 , 0.69)** | ******* |  | **0.24** | **(0.01 , 0.47)** | ***** |
| **61-70** | **15** | **0.45** | **(-0.01 , 0.91)** |  |  | **0.26** | **(-0.26 , 0.79)** |  |  | **0.58** | **(0.10 , 1.06)** | ***** |  | **0.22** | **(-0.28 , 0.72)** |  |  | **0.49** | **(0.00 , 0.98)** | **+** |  | **0.31** | **(-0.21 , 0.83)** |  |
| **71-80** | **16** | **-0.24** | **(-0.67 , 0.20)** |  |  | **0.53** | **(0.02 , 1.03)** | ***** |  | **0.78** | **(0.32 , 1.25)** | ****** |  | **0.73** | **(0.24 , 1.21)** | ****** |  | **0.69** | **(0.22 , 1.16)** | ****** |  | **0.35** | **(-0.15 , 0.85)** |  |
| **81-** | **20** | **-0.06** | **(-0.46 , 0.33)** |  |  | **0.06** | **(-0.39 , 0.51)** |  |  | **0.54** | **(0.12 , 0.95)** | ***** |  | **0.24** | **(-0.20 , 0.67)** |  |  | **0.37** | **(-0.05 , 0.80)** | **+** |  | **0.06** | **(-0.38 , 0.51)** |  |
| **p for linear trend** | | **<0.001 (positive)** | | |  | **<0.001 (positive)** | | |  | **<0.001 (positive)** | | |  | **<0.001 (positive)** | | |  | **<0.001 (positive)** | | |  | **<0.001 (positive)** | | |
| **Middle (aged 30-59 years)** | |  | | |  |  | | |  |  | | |  |  | | |  |  | | |  |  | | |
| **≤20** | **10,896** | **Ref** |  |  |  | **Ref** |  |  |  | **Ref** |  |  |  | **Ref** |  |  |  | **Ref** |  |  |  | **Ref** |  |  |
| **21-30** | **1,735** | **0.08** | **(0.03 , 0.13)** | ****** |  | **0.15** | **(0.10 , 0.20)** | ******* |  | **0.27** | **(0.22 , 0.32)** | ******* |  | **0.20** | **(0.15 , 0.25)** | ******* |  | **0.13** | **(0.08 , 0.17)** | ******* |  | **0.12** | **(0.07 , 0.17)** | ******* |
| **31-40** | **942** | **0.16** | **(0.10 , 0.22)** | ******* |  | **0.24** | **(0.18 , 0.30)** | ******* |  | **0.50** | **(0.44 , 0.57)** | ******* |  | **0.39** | **(0.32 , 0.45)** | ******* |  | **0.19** | **(0.12 , 0.25)** | ******* |  | **0.22** | **(0.16 , 0.29)** | ******* |
| **41-50** | **462** | **0.13** | **(0.05 , 0.22)** | ****** |  | **0.22** | **(0.12 , 0.30)** | ******* |  | **0.53** | **(0.44 , 0.62)** | ******* |  | **0.33** | **(0.24 , 0.42)** | ******* |  | **0.21** | **(0.13 , 0.29)** | ******* |  | **0.18** | **(0.09 , 0.27)** | ******* |
| **51-60** | **240** | **-0.12** | **(-0.23 , 0.00)** | **+** |  | **0.23** | **(0.10 , 0.35)** | ******* |  | **0.57** | **(0.44 , 0.70)** | ******* |  | **0.49** | **(0.37 , 0.62)** | ******* |  | **0.26** | **(0.14 , 0.38)** | ******* |  | **0.24** | **(0.12 , 0.37)** | ******* |
| **61-70** | **110** | **0.05** | **(-0.13 , 0.22)** |  |  | **0.22** | **(0.04 , 0.40)** | ***** |  | **0.84** | **(0.65 , 1.02)** | ******* |  | **0.77** | **(0.59 , 0.95)** | ******* |  | **0.54** | **(0.37 , 0.71)** | ******* |  | **0.41** | **(0.23 , 0.59)** | ******* |
| **71-80** | **81** | **-0.13** | **(-0.33 , 0.07)** |  |  | **0.34** | **(0.13 , 0.55)** | ****** |  | **0.77** | **(0.56 , 0.98)** | ******* |  | **0.68** | **(0.47 , 0.89)** | ******* |  | **0.56** | **(0.36 , 0.76)** | ******* |  | **0.44** | **(0.23 , 0.65)** | ******* |
| **81-** | **69** | **0.05** | **(-0.17 , 0.26)** |  |  | **0.41** | **(0.18 , 0.64)** | ******* |  | **0.64** | **(0.41 , 0.87)** | ******* |  | **0.61** | **(0.38 , 0.84)** | ****** |  | **0.34** | **(0.13 , 0.56)** | ****** |  | **0.13** | **(-0.10 , 0.36)** |  |
| **p for linear trend** | | **<0.001 (positive)** | | |  | **<0.001 (positive)** | | |  | **<0.001 (positive)** | | |  | **<0.001 (positive)** | | |  | **<0.001 (positive)** | | |  | **<0.001 (positive)** | | |
| **Older (aged 60 or older)** | | |  |  |  |  |  |  |  |  |  |  |  |  |  |  |  |  |  |  |  |  |  |  |
| **≤20** | **968** | **Ref** |  |  |  | **Ref** |  |  |  | **Ref** |  |  |  | **Ref** |  |  |  | **Ref** |  |  |  | **Ref** |  |  |
| **21-30** | **93** | **0.17** | **(-0.04 , 0.39)** |  |  | **0.22** | **(0.03 , 0.41)** | ***** |  | **0.27** | **(0.08 , 0.46)** | ****** |  | **0.29** | **(0.11 , 0.47)** | ****** |  | **0.23** | **(0.06 , 0.39)** | ****** |  | **0.08** | **(-0.12 , 0.28)** |  |
| **31-40** | **31** | **-0.16** | **(-0.52 , 0.20)** |  |  | **0.21** | **(-0.11 , 0.53)** |  |  | **0.23** | **(-0.09 , 0.54)** |  |  | **0.21** | **(-0.09 , 0.51)** |  |  | **0.08** | **(-0.20 , 0.36)** |  |  | **0.09** | **(-0.24 , 0.41)** |  |
| **41-50** | **9** | **0.09** | **(-0.56 , 0.75)** |  |  | **0.35** | **(-0.24 , 0.93)** |  |  | **0.31** | **(-0.27 , 0.89)** |  |  | **0.67** | **(0.12 , 1.21)** | ***** |  | **0.45** | **(-0.06 , 0.97)** | **+** |  | **-0.02** | **(-0.62 , 0.58)** |  |
| **51-60** | **5** | **0.99** | **(0.12 , 1.87)** | ***** |  | **1.41** | **(0.63 , 2.19)** | ******* |  | **1.02** | **(0.24 , 1.80)** | ***** |  | **0.71** | **(-0.02 , 1.44)** | **+** |  | **1.08** | **(0.39 , 1.77)** | ****** |  | **0.35** | **(-0.45 , 1.16)** |  |
| **61-70** | **2** | **1.36** | **(-0.02 , 2.74)** | **+** |  | **1.10** | **(-0.13 , 2.33)** | **+** |  | **1.16** | **(-0.07 , 2.38)** |  |  | **0.56** | **(-0.60 , 1.71)** |  |  | **0.51** | **(-0.58 , 1.60)** |  |  | **0.21** | **(-1.06 , 1.48)** |  |
| **71-80** | **0** | **-** |  |  |  | **-** |  |  |  | **-** |  |  |  | **-** |  |  |  | **-** |  |  |  | **-** |  |  |
| **81-** | **2** | **-0.23** | **(-1.61 , 1.16)** |  |  | **-0.37** | **(-1.61 , 0.86)** |  |  | **0.82** | **(-0.41 , 2.05)** |  |  | **-0.18** | **(-1.34 , 0.98)** |  |  | **-0.66** | **(-1.75 , 0.44)** |  |  | **0.32** | **(-0.96 , 1.60)** |  |
| **p for linear trend** | | **n.s.** | | |  | **<0.001 (positive)** | | |  | **<0.001 (positive)** | | |  | **<0.001 (positive)** | | |  | **<0.01 (positive)** | | |  | **n.s.** | | |
| **Interaction between overtime and age-category** | | | | |  |  |  |  |  |  |  |  |  |  |  |  |  |  |  |  |  |  |  |  |
| **Middle (ref) versus Young** | | |  |  |  |  |  |  |  |  |  |  |  |  |  |  |  |  |  |  |  |  |  |  |
| **21-30** |  | **-0.05** | **(-0.15 , 0.06)** |  |  | **-0.02** | **(-0.13 , 0.09)** |  |  | **-0.05** | **(-0.16 , 0.07)** |  |  | **-0.14** | **(-0.25 , -0.03)** | ***** |  | **-0.03** | **(-0.13 , 0.07)** |  |  | **-0.03** | **(-0.14 , 0.08)** | ***** |
| **31-40** |  | **-0.01** | **(-0.15 , 0.12)** |  |  | **-0.08** | **(-0.23 , 0.06)** |  |  | **-0.15** | **(-0.29 , 0.00)** | ***** |  | **-0.16** | **(-0.30 , -0.01)** | ***** |  | **0.06** | **(-0.08 , 0.20)** |  |  | **-0.15** | **(-0.29 , 0.00)** |  |
| **41-50** |  | **0.00** | **(-0.20 , 0.21)** |  |  | **-0.14** | **(-0.36 , 0.08)** |  |  | **-0.07** | **(-0.29 , 0.15)** |  |  | **-0.02** | **(-0.24 , 0.20)** |  |  | **0.01** | **(-0.20 , 0.21)** |  |  | **-0.06** | **(-0.28 , 0.16)** |  |
| **51-60** |  | **0.27** | **(0.04 , 0.51)** | ***** |  | **-0.15** | **(-0.40 , 0.10)** |  |  | **0.09** | **(-0.16 , 0.34)** |  |  | **-0.06** | **(-0.30 , 0.19)** |  |  | **0.20** | **(-0.03 , 0.44)** | **+** |  | **0.05** | **(-0.20 , 0.29)** |  |
| **61-70** |  | **0.39** | **(-0.11 , 0.89)** |  |  | **0.00** | **(-0.52 , 0.53)** |  |  | **-0.24** | **(-0.76 , 0.29)** |  |  | **-0.54** | **(-1.06 , -0.03)** | ***** |  | **-0.11** | **(-0.60 , 0.39)** |  |  | **-0.05** | **(-0.58 , 0.47)** |  |
| **71-80** |  | **-0.07** | **(-0.56 , 0.42)** |  |  | **0.18** | **(-0.34 , 0.70)** |  |  | **0.06** | **(-0.47 , 0.58)** |  |  | **0.09** | **(-0.42 , 0.61)** |  |  | **0.18** | **(-0.31 , 0.67)** |  |  | **-0.04** | **(-0.56 , 0.49)** |  |
| **81-** |  | **-0.12** | **(-0.58 , 0.34)** |  |  | **-0.35** | **(-0.84 , 0.13)** |  |  | **-0.09** | **(-0.58 , 0.39)** |  |  | **-0.35** | **(-0.83 , 0.12)** |  |  | **0.03** | **(-0.43 , 0.48)** |  |  | **-0.04** | **(-0.52 , 0.45)** |  |
| **Middle (ref) versus Older** | | |  |  |  |  |  |  |  |  |  |  |  |  |  |  |  |  |  |  |  |  |  |  |
| **21-30** |  | **0.12** | **(-0.08 , 0.32)** |  |  | **0.10** | **(-0.11 , 0.31)** |  |  | **0.06** | **(-0.15 , 0.27)** |  |  | **0.13** | **(-0.08 , 0.33)** |  |  | **0.14** | **(-0.06 , 0.33)** |  |  | **0.01** | **(-0.20 , 0.22)** |  |
| **31-40** |  | **-0.27** | **(-0.60 , 0.06)** |  |  | **0.01** | **(-0.35 , 0.36)** |  |  | **-0.20** | **(-0.55 , 0.16)** |  |  | **-0.14** | **(-0.48 , 0.21)** |  |  | **-0.08** | **(-0.41 , 0.25)** |  |  | **-0.07** | **(-0.42 , 0.29)** |  |
| **41-50** |  | **0.02** | **(-0.59 , 0.63)** |  |  | **0.17** | **(-0.48 , 0.81)** |  |  | **-0.14** | **(-0.78 , 0.50)** |  |  | **0.34** | **(-0.29 , 0.97)** |  |  | **0.26** | **(-0.35 , 0.86)** |  |  | **-0.12** | **(-0.76 , 0.52)** |  |
| **51-60** |  | **1.28** | **(0.47 , 2.10)** | ****** |  | **1.32** | **(0.46 , 2.18)** | ****** |  | **0.65** | **(-0.21 , 1.50)** |  |  | **0.31** | **(-0.53 , 1.16)** |  |  | **0.89** | **(0.08 , 1.70)** | ***** |  | **0.28** | **(-0.58 , 1.14)** |  |
| **61-70** |  | **1.33** | **(0.04 , 2.61)** | ***** |  | **0.82** | **(-0.54 , 2.18)** |  |  | **0.34** | **(-1.01 , 1.69)** |  |  | **-0.25** | **(-1.59 , 1.08)** |  |  | **-0.01** | **(-1.28 , 1.26)** |  |  | **-0.13** | **(-1.48 , 1.23)** |  |
| **71-80** |  | **-** |  |  |  | **-** |  |  |  | **-** |  |  |  | **-** |  |  |  | **-** |  |  |  | **-** |  |  |
| **81-** |  | **-0.36** | **(-1.65 , 0.93)** |  |  | **-0.55** | **(-1.92 , 0.82)** |  |  | **0.32** | **(-1.04 , 1.68)** |  |  | **-0.66** | **(-2.01 , 0.68)** |  |  | **-0.81** | **(-2.09 , 0.47)** |  |  | **0.31** | **(-1.05 , 1.67)** |  |

+ p<0.1, *p<0.05, ** p<0.01, ***p<0.001, Higher score indicates unfavorable stress-response. All betas were adjusted by age, type of job, job class, employment status, type of schedule, company size, company industry, job control, supervisors support and coworker’s support. Lack of vigor was derived by reversing the score of vigor for harmonization with other stress-response scales, which higher score indicates unfavorable stress-response

| **Table 4. Association between overtime work hours and stress responses ; results of multiple linear regression stratified by gender and job-class** | | | | | | | | | | | | | | | | | | | | | | | | |
| --- | --- | --- | --- | --- | --- | --- | --- | --- | --- | --- | --- | --- | --- | --- | --- | --- | --- | --- | --- | --- | --- | --- | --- | --- |
| **Overtime work**  **hours/month** | **n** | **Lack of Vigor** | | |  | **Irritability** | | |  | **Fatigue** | | |  | **Anxiety** | | |  | **Depression** | | |  | **Somatic responses** | | |
|  |  | **beta** | **95％ CI** | **p** |  | **beta** | **95％ CI** | **p** |  | **beta** | **95％ CI** | **p** |  | **beta** | **95％ CI** | **p** |  | **beta** | **95％ CI** | **p** |  | **beta** | **95％ CI** | **p** |
| **Men** |  |  |  |  |  |  |  |  |  |  |  |  |  |  |  |  |  |  |  |  |  |  |  |  |
| **Workers in managerial or directorial position** | | | |  |  |  |  |  |  |  |  |  |  |  |  |  |  |  |  |  |  |  |  |  |
| **≤20** | **2,117** | **Ref** |  |  |  | **Ref** |  |  |  | **Ref** |  |  |  | **Ref** |  |  |  | **Ref** |  |  |  | **Ref** |  |  |
| **21-30** | **1,602** | **0.07** | **(0.01 , 0.12)** | ***** |  | **0.11** | **(0.06 , 0.17)** | ******* |  | **0.15** | **(0.10 , 0.21)** | ******* |  | **0.14** | **(0.08 , 0.19)** | ******* |  | **0.08** | **(0.02 , 0.13)** | ****** |  | **0.04** | **(-0.02 , 0.09)** |  |
| **31-40** | **1,639** | **0.02** | **(-0.04 , 0.07)** |  |  | **0.12** | **(0.06 , 0.18)** | ******* |  | **0.27** | **(0.21 , 0.32)** | ******* |  | **0.24** | **(0.18 , 0.29)** | ******* |  | **0.13** | **(0.07 , 0.18)** | ******* |  | **0.07** | **(0.01 , 0.12)** | ***** |
| **41-50** | **1,475** | **-0.03** | **(-0.09 , 0.03)** |  |  | **0.17** | **(0.11 , 0.23)** | ******* |  | **0.31** | **(0.25 , 0.37)** | ******* |  | **0.28** | **(0.22 , 0.34)** | ******* |  | **0.11** | **(0.05 , 0.17)** | ******* |  | **0.06** | **(-0.01 , 0.12)** | **+** |
| **51-60** | **1,339** | **-0.03** | **(-0.09 , 0.04)** |  |  | **0.19** | **(0.12 , 0.25)** | ******* |  | **0.39** | **(0.33 , 0.45)** | ******* |  | **0.32** | **(0.25 , 0.38)** | ******* |  | **0.12** | **(0.06 , 0.18)** | ******* |  | **0.08** | **(0.02 , 0.15)** | ***** |
| **61-70** | **832** | **-0.12** | **(-0.19 , -0.05)** | ****** |  | **0.16** | **(0.09 , 0.23)** | ******* |  | **0.43** | **(0.35 , 0.50)** | ******* |  | **0.33** | **(0.26 , 0.40)** | ******* |  | **0.09** | **(0.02 , 0.16)** | ****** |  | **0.02** | **(-0.06 , 0.09)** |  |
| **71-80** | **915** | **-0.08** | **(-0.15 , -0.01)** | ***** |  | **0.31** | **(0.24 , 0.38)** | ******* |  | **0.56** | **(0.49 , 0.63)** | ******* |  | **0.47** | **(0.40 , 0.54)** | ******* |  | **0.18** | **(0.11 , 0.25)** | ******* |  | **0.13** | **(0.06 , 0.21)** | ******* |
| **81-** | **430** | **-0.02** | **(-0.11 , 0.07)** |  |  | **0.33** | **(0.24 , 0.42)** | ******* |  | **0.72** | **(0.63 , 0.81)** | ******* |  | **0.47** | **(0.38 , 0.56)** | ******* |  | **0.23** | **(0.14 , 0.32)** | ******* |  | **0.11** | **(0.01 , 0.20)** | ***** |
| **p for linear trend** | | **<0.001 (negative)** | | |  | **<0.001 (positive)** | | |  | **<0.001 (positive)** | | |  | **<0.001 (positive)** | | |  | **<0.001 (positive)** | | |  | **<0.001 (positive)** | | |
| **Workers in regular position** | | |  |  |  |  |  |  |  |  |  |  |  |  |  |  |  |  |  |  |  |  |  |  |
| **≤20** | **13,634** | **Ref** |  |  |  | **Ref** |  |  |  | **Ref** |  |  |  | **Ref** |  |  |  | **Ref** |  |  |  | **Ref** |  |  |
| **21-30** | **5,658** | **0.04** | **(0.01 , 0.07)** | ***** |  | **0.12** | **(0.09 , 0.15)** | ******* |  | **0.17** | **(0.14 , 0.20)** | ******* |  | **0.10** | **(0.07 , 0.13)** | ******* |  | **0.04** | **(0.01 , 0.07)** | ****** |  | **0.02** | **(-0.01 , 0.05)** |  |
| **31-40** | **4,173** | **0.01** | **(-0.02 , 0.04)** |  |  | **0.16** | **(0.13 , 0.19)** | ******* |  | **0.27** | **(0.24 , 0.30)** | ******* |  | **0.13** | **(0.10 , 0.16)** | ******* |  | **0.04** | **(0.01 , 0.08)** | ***** |  | **0.07** | **(0.03 , 0.10)** | ******* |
| **41-50** | **2,622** | **0.02** | **(-0.02 , 0.06)** |  |  | **0.16** | **(0.12 , 0.20)** | ******* |  | **0.41** | **(0.37 , 0.44)** | ******* |  | **0.20** | **(0.16 , 0.24)** | ******* |  | **0.14** | **(0.10 , 0.18)** | ******* |  | **0.17** | **(0.13 , 0.21)** | ******* |
| **51-60** | **2,155** | **-0.04** | **(-0.08 , 0.00)** | **+** |  | **0.18** | **(0.14 , 0.23)** | ******* |  | **0.44** | **(0.40 , 0.49)** | ******* |  | **0.22** | **(0.18 , 0.27)** | ******* |  | **0.12** | **(0.08 , 0.16)** | ******* |  | **0.16** | **(0.11 , 0.20)** | ******* |
| **61-70** | **982** | **-0.13** | **(-0.19 , -0.07)** | ******* |  | **0.23** | **(0.16 , 0.29)** | ******* |  | **0.55** | **(0.49 , 0.61)** | ******* |  | **0.41** | **(0.35 , 0.47)** | ******* |  | **0.26** | **(0.19 , 0.32)** | ******* |  | **0.26** | **(0.20 , 0.32)** | ******* |
| **71-80** | **825** | **-0.18** | **(-0.24 , -0.11)** | ******* |  | **0.25** | **(0.18 , 0.31)** | ******* |  | **0.61** | **(0.54 , 0.67)** | ******* |  | **0.42** | **(0.36 , 0.49)** | ******* |  | **0.31** | **(0.24 , 0.38)** | ******* |  | **0.30** | **(0.23 , 0.37)** | ******* |
| **81-** | **366** | **0.01** | **(-0.08 , 0.10)** |  |  | **0.42** | **(0.33 , 0.52)** | ******* |  | **0.76** | **(0.67 , 0.86)** | ******* |  | **0.37** | **(0.27 , 0.47)** | ******* |  | **0.31** | **(0.21 , 0.41)** | ******* |  | **0.34** | **(0.24 , 0.44)** | ******* |
| **p for linear trend** | | **<0.001 (negative)** | | |  | **<0.001 (positive)** | | |  | **<0.001 (positive)** | | |  | **<0.001 (positive)** | | |  | **<0.001 (positive)** | | |  | **<0.001 (positive)** | | |
| **Interaction between overtime and age-category** | | |  |  |  |  |  |  |  |  |  |  |  |  |  |  |  |  |  |  |  |  |  |  |
| **Regular (ref) versus Manager** | | |  |  |  |  |  |  |  |  |  |  |  |  |  |  |  |  |  |  |  |  |  |  |
| **21-30** |  | **0.03** | **(-0.03 , 0.09)** |  |  | **0.00** | **(-0.07 , 0.06)** |  |  | **-0.01** | **(-0.07 , 0.06)** |  |  | **0.03** | **(-0.03 , 0.10)** |  |  | **0.02** | **(-0.04 , 0.09)** |  |  | **0.02** | **(-0.05 , 0.08)** |  |
| **31-40** |  | **0.00** | **(-0.06 , 0.07)** |  |  | **-0.02** | **(-0.09 , 0.04)** |  |  | **0.01** | **(-0.06 , 0.07)** |  |  | **0.10** | **(0.04 , 0.17)** | ****** |  | **0.07** | **(0.00 , 0.14)** | ***** |  | **0.01** | **(-0.06 , 0.07)** |  |
| **41-50** |  | **-0.05** | **(-0.12 , 0.02)** |  |  | **0.02** | **(-0.05 , 0.09)** |  |  | **-0.09** | **(-0.16 , -0.02)** | ***** |  | **0.07** | **(0.00 , 0.14)** | ***** |  | **-0.06** | **(-0.13 , 0.02)** |  |  | **-0.10** | **(-0.17 , -0.02)** | ****** |
| **51-60** |  | **0.01** | **(-0.06 , 0.08)** |  |  | **0.04** | **(-0.04 , 0.11)** |  |  | **-0.04** | **(-0.11 , 0.03)** |  |  | **0.08** | **(0.00 , 0.15)** | ***** |  | **-0.04** | **(-0.11 , 0.04)** |  |  | **-0.04** | **(-0.12 , 0.03)** |  |
| **61-70** |  | **0.00** | **(-0.09 , 0.09)** |  |  | **-0.03** | **(-0.12 , 0.07)** |  |  | **-0.11** | **(-0.20 , -0.02)** | ***** |  | **-0.10** | **(-0.19 , 0.00)** | ***** |  | **-0.19** | **(-0.29 , -0.10)** | ******* |  | **-0.22** | **(-0.31 , -0.12)** | ******* |
| **71-80** |  | **0.09** | **(0.00 , 0.18)** | **+** |  | **0.10** | **(0.01 , 0.20)** | ***** |  | **-0.04** | **(-0.13 , 0.05)** |  |  | **0.02** | **(-0.08 , 0.11)** |  |  | **-0.16** | **(-0.26 , -0.07)** | ****** |  | **-0.13** | **(-0.23 , -0.03)** | ****** |
| **81-** |  | **0.01** | **(-0.12 , 0.14)** |  |  | **-0.09** | **(-0.22 , 0.05)** |  |  | **-0.04** | **(-0.17 , 0.10)** |  |  | **0.09** | **(-0.04 , 0.22)** |  |  | **-0.10** | **(-0.23 , 0.03)** |  |  | **-0.23** | **(-0.37 , -0.09)** |  |
| **Women** |  |  |  |  |  |  |  |  |  |  |  |  |  |  |  |  |  |  |  |  |  |  |  |  |
| **Workers in managerial or directorial position** | | | |  |  |  |  |  |  |  |  |  |  |  |  |  |  |  |  |  |  |  |  |  |
| **≤20** | **151** | **Ref** |  |  |  | **Ref** |  |  |  | **Ref** |  |  |  | **Ref** |  |  |  | **Ref** |  |  |  | **Ref** |  |  |
| **21-30** | **124** | **0.08** | **(-0.15 , 0.31)** |  |  | **0.04** | **(-0.18 , 0.25)** |  |  | **0.04** | **(-0.19 , 0.27)** |  |  | **-0.01** | **(-0.23 , 0.21)** |  |  | **0.02** | **(-0.19 , 0.23)** |  |  | **0.04** | **(-0.18 , 0.26)** |  |
| **31-40** | **125** | **0.08** | **(-0.16 , 0.31)** |  |  | **0.23** | **(0.01 , 0.45)** | ***** |  | **0.43** | **(0.20 , 0.67)** | ******* |  | **0.48** | **(0.26 , 0.71)** | ******* |  | **0.17** | **(-0.05 , 0.38)** |  |  | **0.28** | **(0.05 , 0.50)** | ***** |
| **41-50** | **81** | **0.23** | **(-0.04 , 0.49)** | **+** |  | **0.09** | **(-0.16 , 0.34)** |  |  | **0.35** | **(0.09 , 0.62)** | ****** |  | **0.33** | **(0.07 , 0.58)** | ***** |  | **0.03** | **(-0.21 , 0.27)** |  |  | **0.10** | **(-0.16 , 0.35)** |  |
| **51-60** | **64** | **0.01** | **(-0.28 , 0.31)** |  |  | **0.10** | **(-0.17 , 0.37)** |  |  | **0.39** | **(0.09 , 0.68)** | ****** |  | **0.28** | **(0.00 , 0.56)** | **+** |  | **0.03** | **(-0.23 , 0.30)** |  |  | **0.14** | **(-0.14 , 0.43)** |  |
| **61-70** | **33** | **0.43** | **(0.06 , 0.80)** | ***** |  | **0.46** | **(0.11 , 0.80)** | ******* |  | **0.86** | **(0.49 , 1.23)** | ******* |  | **0.85** | **(0.50 , 1.21)** | ******* |  | **0.39** | **(0.06 , 0.72)** | ****** |  | **0.28** | **(-0.08 , 0.64)** |  |
| **71-80** | **18** | **0.10** | **(-0.38 , 0.57)** |  |  | **0.23** | **(-0.22 , 0.67)** |  |  | **1.16** | **(0.68 , 1.63)** | ******* |  | **0.70** | **(0.25 , 1.15)** | ****** |  | **0.77** | **(0.34 , 1.19)** | ******* |  | **0.66** | **(0.20 , 1.12)** | ****** |
| **81-** | **20** | **-0.04** | **(-0.49 , 0.40)** |  |  | **0.22** | **(-0.20 , 0.63)** |  |  | **0.52** | **(0.07 , 0.97)** | ***** |  | **0.43** | **(0.00 , 0.86)** | ***** |  | **0.09** | **(-0.31 , 0.50)** |  |  | **0.20** | **(-0.24 , 0.63)** |  |
| **p for linear trend** | | **n.s.** | | |  | **<0.001 (positive)** | | |  | **<0.001 (positive)** | | |  | **<0.001 (positive)** | | |  | **<0.05 (positive)** | | |  | **<0.05 (positive)** | | |
| **Workers in regular position** | | |  |  |  |  |  |  |  |  |  |  |  |  |  |  |  |  |  |  |  |  |  |  |
| **≤20** | **13,378** | **Ref** |  |  |  | **Ref** |  |  |  | **Ref** |  |  |  | **Ref** |  |  |  | **Ref** |  |  |  | **Ref** |  |  |
| **21-30** | **2,177** | **0.07** | **(0.03 , 0.12)** | ****** |  | **0.15** | **(0.11 , 0.19)** | ******* |  | **0.27** | **(0.23 , 0.32)** | ******* |  | **0.20** | **(0.15 , 0.24)** | ******* |  | **0.14** | **(0.09 , 0.18)** | ******* |  | **0.12** | **(0.08 , 0.17)** | ******* |
| **31-40** | **1,094** | **0.16** | **(0.10 , 0.22)** | ******* |  | **0.22** | **(0.16 , 0.28)** | ******* |  | **0.47** | **(0.41 , 0.53)** | ******* |  | **0.35** | **(0.29 , 0.40)** | ******* |  | **0.20** | **(0.14 , 0.26)** | ******* |  | **0.19** | **(0.13 , 0.25)** | ******* |
| **41-50** | **486** | **0.13** | **(0.04 , 0.21)** | ****** |  | **0.22** | **(0.13 , 0.30)** | ******* |  | **0.54** | **(0.45 , 0.63)** | ******* |  | **0.34** | **(0.25 , 0.42)** | ******* |  | **0.24** | **(0.16 , 0.33)** | ******* |  | **0.20** | **(0.12 , 0.29)** | ******* |
| **51-60** | **262** | **-0.05** | **(-0.17 , 0.06)** |  |  | **0.22** | **(0.10 , 0.34)** | ******* |  | **0.63** | **(0.51 , 0.75)** | ******* |  | **0.55** | **(0.43 , 0.66)** | ******* |  | **0.39** | **(0.28 , 0.51)** | ******* |  | **0.29** | **(0.17 , 0.41)** | ******* |
| **61-70** | **94** | **0.03** | **(-0.16 , 0.22)** |  |  | **0.16** | **(-0.04 , 0.36)** |  |  | **0.83** | **(0.63 , 1.02)** | ******* |  | **0.66** | **(0.46 , 0.85)** | ******* |  | **0.57** | **(0.39 , 0.76)** | ******* |  | **0.48** | **(0.29 , 0.68)** | ******* |
| **71-80** | **79** | **-0.19** | **(-0.39 , 0.02)** | **+** |  | **0.40** | **(0.18 , 0.61)** | ******* |  | **0.70** | **(0.48 , 0.91)** | ******* |  | **0.70** | **(0.48 , 0.91)** | ******* |  | **0.55** | **(0.35 , 0.75)** | ******* |  | **0.39** | **(0.18 , 0.61)** | ******* |
| **81-** | **71** | **0.05** | **(-0.17 , 0.26)** |  |  | **0.33** | **(0.10 , 0.55)** | ****** |  | **0.64** | **(0.42 , 0.87)** | ******* |  | **0.53** | **(0.31 , 0.75)** | ******* |  | **0.38** | **(0.17 , 0.60)** | ******* |  | **0.13** | **(-0.10 , 0.35)** |  |
| **p for linear trend** | | **<0.001 (positive)** | | |  | **<0.001 (positive)** | | |  | **<0.001 (positive)** | | |  | **<0.001 (positive)** | | |  | **<0.001 (positive)** | | |  | **<0.001 (positive)** | | |
| **Interaction between overtime and age-category** | | |  |  |  |  |  |  |  |  |  |  |  |  |  |  |  |  |  |  |  |  |  |  |
| **Regular (ref) versus Manager** | | |  |  |  |  |  |  |  |  |  |  |  |  |  |  |  |  |  |  |  |  |  |  |
| **21-30** |  | **-0.03** | **(-0.25 , 0.19)** |  |  | **-0.11** | **(-0.34 , 0.13)** |  |  | **-0.30** | **(-0.53 , -0.07)** | ***** |  | **-0.23** | **(-0.46 , 0.00)** | **+** |  | **-0.15** | **(-0.37 , 0.07)** |  |  | **-0.09** | **(-0.33 , 0.14)** |  |
| **31-40** |  | **-0.14** | **(-0.37 , 0.09)** |  |  | **0.02** | **(-0.22 , 0.26)** |  |  | **-0.13** | **(-0.36 , 0.11)** |  |  | **0.12** | **(-0.12 , 0.35)** |  |  | **-0.06** | **(-0.28 , 0.16)** |  |  | **0.05** | **(-0.19 , 0.28)** |  |
| **41-50** |  | **0.06** | **(-0.20 , 0.33)** |  |  | **-0.14** | **(-0.41 , 0.14)** |  |  | **-0.25** | **(-0.52 , 0.03)** | **+** |  | **-0.03** | **(-0.30 , 0.24)** |  |  | **-0.24** | **(-0.49 , 0.02)** | **+** |  | **-0.15** | **(-0.43 , 0.12)** |  |
| **51-60** |  | **0.01** | **(-0.28 , 0.31)** |  |  | **-0.07** | **(-0.37 , 0.24)** |  |  | **-0.31** | **(-0.61 , 0.00)** | ***** |  | **-0.28** | **(-0.58 , 0.02)** | **+** |  | **-0.40** | **(-0.68 , -0.11)** | ****** |  | **-0.19** | **(-0.49 , 0.12)** |  |
| **61-70** |  | **0.29** | **(-0.11 , 0.68)** |  |  | **0.29** | **(-0.12 , 0.71)** |  |  | **-0.14** | **(-0.55 , 0.28)** |  |  | **0.14** | **(-0.27 , 0.55)** |  |  | **-0.23** | **(-0.62 , 0.16)** |  |  | **-0.29** | **(-0.71 , 0.12)** |  |
| **71-80** |  | **0.19** | **(-0.31 , 0.68)** |  |  | **-0.09** | **(-0.61 , 0.43)** |  |  | **0.35** | **(-0.17 , 0.86)** |  |  | **0.02** | **(-0.49 , 0.53)** |  |  | **0.19** | **(-0.29 , 0.68)** |  |  | **0.26** | **(-0.26 , 0.78)** |  |
| **81-** |  | **-0.20** | **(-0.68 , 0.28)** |  |  | **-0.09** | **(-0.60 , 0.42)** |  |  | **-0.19** | **(-0.70 , 0.31)** |  |  | **-0.09** | **(-0.58 , 0.41)** |  |  | **-0.30** | **(-0.77 , 0.17)** |  |  | **0.03** | **(-0.48 , 0.53)** |  |

+ p<0.1, *p<0.05, ** p<0.01, ***p<0.001, Higher score indicates unfavorable stress-response. All betas were adjusted by age, type of job, employment status, type of schedule, company size, company industry, job control, supervisors support and coworker’s support. Lack of vigor was derived by reversing the score of vigor for harmonization with other stress-response scales, which higher score indicates unfavorable stress-response

***Comment #2***

**On a related note, you seem to provide a more fine-grained perspective on hours of overtime. Are there any inconsistencies that would suggest taking a closer look? In other words, besides the technical aspect: How does your approach advance knowledge? Does it provide a more accurate picture? Does it help to gain a more integrated picture (comparing many different types of strain in one study)? Does it facilitate comparison between strain indicators? Does it add data on Japanese employees? I recommend stating more clearly how this study enriches existing research. I am quite sure it does.**

(Our response to this comment)

We thank for this important suggestion. As explained in previous our response for comment #1, we could conduct additional stratified analyses, which were not sufficiently performed in past studies. Especially in Japan, there has been an extensive debate regarding the upper overtime-limit which has been raised to 100 hours/month. Therefore, detailed epidemiological data with this stratified analysis is beneficial for the presentation of a nuanced picture of the relationship between overtime and stress response. To clarify these points, we revised the introduction part. We appreciate your understanding.

(Introduction section, page 5-6, line 68-78)

*In this context, it is important to consider the upper overtime-limit to prevent mental illness among workers. So far, several studies have investigated associations between long working hours and mental health (1,14). However, in many studies used tertile (15,16), quartile (17–19) or quintile (20,21) cut-off were used for evaluating effects of amount of overtime work.. Studies in which a broader range of overtime is used are needed for the establishment of an upper overtime-limit as a health policy. In addition, there are only few published studies in which gender-stratified analysis has been performed. This is due to the limited number of women with long working hours (19,22). Labor force participation among women of working age increased substantially worldwide (23). Furthermore, occupational class might influence the relation between overtime and health outcome (24). To nuance the picture of associations between overtime and mental health, stratified analysis by these factors is also needed.*

***Comment #3***

**The choice of strain measures is not justified at all. They seem to be taken for granted. However, it is not clear why exactly those aspects were chosen. I understand that this has been discussed in the other study using these data. For readers who do not want to take the effort of looking up details elsewhere, please add a short description of the strain indicators. What characterizes fatigue in the sense you mean? How does it differ from lack of vigor both conceptually and empirically? Is “lack of vigor” the opposite of vigor? I found the expression quite complicated, as it includes a negation. Please also note that there is a long list of papers on the question whether vigor is the opposite of exhaustion (work engagement vs. burnout) (Demerouti, Mostert, & Bakker, 2010; González-Romá, Schaufeli, Bakker, & Lloret, 2006).**

(Our response for this comment)

We appreciate this comment. First of all, we apologize for this confusion. In this study, we calculated the “lack of vigor” score by reversing the “vigor” score in the questionnaire, in order to harmonize with the other stress-response scales, in which higher score means unfavorable state. To avoid this confusion, we added footnotes in all tables and figures.

The BJSQ was developed by referring the Profile Of Mood States (POMS), the State-Trail Anxiety Inventory (STAI) and the Center for epidemiologic Studies for Depression Scale (CES-D), Screener for the Somatoform Disorders and the Subjective Wellbeing Inventory (SUBI). In BJSQ, vigor was assessed by three questions (Response categories 1: I have been very active, 2:I have been full of energy, 3 I have been lively). Fatigue was also assessed also by means of three items (1:I have felt extremely tired, 2:I have felt exhausted, 3:I have felt weary or listless). To clarify this information, we revised Materials and methods section, table footnotes, and also added detailed BJSQ items in Supplemental table 1-1.

Finally, we calculated the correlation coefficients among six strain measures. As shown in the table below, “lack of vigor” (reverse score of vigor) and “fatigue” are not strongly correlated (r=0.341). We added this table in Supplemental table 1-2.

(Materials and methods section, page 9, line 130-134)

*Stress responses were assessed using the Brief Job Stress Questionnaire (BJSQ) following the recommended protocol of the Stress Check Program (25-27).* *The BJSQ was developed by referring the Profile Of Mood States (POMS), the State-Trail Anxiety Inventory (STAI), the Center for epidemiologic Studies for Depression Scale (CES-D), Screener for the Somatoform Disorders and the Subjective Wellbeing Inventory (SUBI) (28).*

(Tables and Figures, footnotes)

*+ p<0.1, *p<0.05, ** p<0.01, ***p<0.001, Higher score indicates unfavorable stress-response. All beta coefficients were adjusted by age, type of job, job class, employment status, type of schedule, company size, company industry, job control, supervisors support and coworker’s support. Lack of vigor was derived from the reversed questionnaire score of vigor for harmonization with other stress-response scales; in these scale a higher score indicates an unfavorable stress-response*

| Supplemental table1-1: Specific items with internal consistency of the scales of Brief Job Stress Questionnaire used in the study | | | | | | |
| --- | --- | --- | --- | --- | --- | --- |
| Category | Scale | Number of item | Question | Mean | SD | Cronbach's alpha |
| Stressor |  |  | *Please answer the following questions concerning your job by circling the number that best fits your situation. (1:Very much so, 2: Moderately so, 3: Somewhat 4: Not at all)* | - | - | - |
|  | Job control | 3 | *I can work at my own pace* | 2.64 | 0.85 | 0.712 |
|  |  |  | *I can choose how and in what order to do my work* | 2.30 | 0.80 |  |
|  |  |  | *I can reflect my opinions on workplace policy* | 2.48 | 0.79 |  |
| Stress-response | |  | *Please answer the following questions concerning your health during the past month by circling the number that best fits your situation. (1:Almost never, 2: Sometimes 3: Often 4:Almost always)* | - | - | - |
|  | Vigor | 3 | *I have been very active* | 2.15 | 0.80 | 0.911 |
|  |  |  | *I have been full of energy* | 2.21 | 0.83 |  |
|  |  |  | *I have been lively* | 2.14 | 0.82 |  |
|  | Irritability | 3 | *I have felt angry* | 2.08 | 0.81 | 0.892 |
|  |  |  | *I have been inwardly annoyed or aggravated* | 2.06 | 0.84 |  |
|  |  |  | *I have felt irritable* | 2.10 | 0.82 |  |
|  | Fatigue | 3 | *I have felt extremely tired* | 2.40 | 0.86 | 0.873 |
|  |  |  | *I have felt exhausted* | 2.04 | 0.92 |  |
|  |  |  | *I have felt weary or listless* | 2.15 | 0.89 |  |
|  | Anxiety | 3 | *I have felt tense* | 2.34 | 0.88 | 0.764 |
|  |  |  | *I have felt worried or insecure* | 2.15 | 0.93 |  |
|  |  |  | *I have felt restless* | 1.80 | 0.84 |  |
|  | Depression | 6 | *I have been depressed* | 1.93 | 0.90 | 0.885 |
|  |  |  | *I have thought that doing anything was a hassle* | 1.76 | 0.81 |  |
|  |  |  | *I have been unable to concentrate* | 1.70 | 0.73 |  |
|  |  |  | *I have felt gloomy* | 1.96 | 0.85 |  |
|  |  |  | *I have been unable to handle work* | 1.42 | 0.64 |  |
|  |  |  | *I have felt sad* | 1.47 | 0.74 |  |
|  | Somatic responses | 11 | *I have felt dizzy* | 1.34 | 0.64 | 0.841 |
|  |  |  | *I have experienced joint pains* | 1.43 | 0.73 |  |
|  |  |  | *I have experienced headaches* | 1.70 | 0.81 |  |
|  |  |  | *I have had a stiff neck and / or shoulders* | 2.41 | 1.05 |  |
|  |  |  | *I have had lower back pain* | 1.94 | 0.96 |  |
|  |  |  | *I have had eyestrain* | 2.52 | 0.95 |  |
|  |  |  | *I have experienced heart palpitations or shortness of breath* | 1.32 | 0.63 |  |
|  |  |  | *I have experienced stomach and / or intestine problems* | 1.63 | 0.81 |  |
|  |  |  | *I have lost my appetite* | 1.31 | 0.60 |  |
|  |  |  | *I have experienced diarrhea and / or constipation* | 1.74 | 0.89 |  |
|  |  |  | *I haven’t been able to sleep well* | 1.66 | 0.84 |  |
| Support |  |  | *Please answer the following questions concerning people around you by circling the number that best fits your situation. (1: Extremely, 2:Very much, 3:Somewhat, 4:Not at all)* | - | - | - |
|  | Supervisor’s support | 3 | *How freely can you talk with your supervisor?* | 2.42 | 0.81 | 0.820 |
|  |  |  | *How reliable are your supervisor when you are troubled?* | 2.30 | 0.84 |  |
|  |  |  | *How well will your supervisor listen to you when you ask for advice on personal matters?* | 2.53 | 0.87 |  |
|  | Coworker’s support | 3 | *How freely can you talk with your coworker?* | 2.07 | 0.76 | 0.793 |
|  |  |  | *How reliable are your coworker when you are troubled?* | 2.26 | 0.78 |  |
|  |  |  | *How well will your coworker listen to you when you ask for advice on personal matters?* | 2.47 | 0.82 |  |

| Supplemental table 1-2: Correlation coefficient between stress response scales in BJSQ | | | | | | |
| --- | --- | --- | --- | --- | --- | --- |
|  | Lack of vigor | Irritability | Fatigue | Anxiety | Depression | Somatic responses |
| Lack of vigor | 1 |  |  |  |  |  |
| Irritability | 0.280*** | 1 |  |  |  |  |
| Fatigue | 0.341*** | 0.515*** | 1 |  |  |  |
| Anxiety | 0.281*** | 0.509*** | 0.623*** | 1 |  |  |
| Depression | 0.429*** | 0.553*** | 0.628*** | 0.704*** | 1 |  |
| Somatic responses | 0.298*** | 0.416*** | 0.581*** | 0.480*** | 0.583*** | 1 |

****p<0.001*

*Lack of vigor was derived by reversing the score of vigor for harmonization with other stress-response scales, which higher score indicates unfavorable stress-response*

***Comment #4***

**Please also add a more systematic review of how overtime is related to fatigue, lack of vigor, depression etc. respectively. These details would help judge your results in the light of prior research. Is irritability the same thing as irritation (Mohr, Müller, Rigotti, Aycan, & Tschan, 2006)? I think, it is important to locate all variables in the nomological network and to be clear what these concepts actually mean.**

(Our response for this comment)

We thank you for this comment. In response to the previous comment (#3), we added explanation how BJSQ was developed in the manuscript. In addition, specific items of BJSQ were added in Supplemental table 1-1. We expect that these additional materials contribute for readers to capture how each scale in BJSQ is measured.

We read the reference you suggested. In that reference, the irritation scale (8-item) responses were classified into cognitive and emotional irritation respectively. The irritability scale in this BJSQ is partly from POMS, so it seems to be close to emotional irritation in the reference.

***Comment #5***

**Methods**

**Please report sample items for each scale. Please also describe the scope of each scale.**

(Our response for this comment)

We appreciate this comment. In response to a similar suggestion from the other reviewer, we added the BJSQ items with detailed information including specific questions and Cronbach’s alpha as a Supplemental table (Supplemental table 1).

***Comment #6***

**You excluded participants from small organizations and participants who did not receive the program? What does receiving the program mean? It is not trivial to me why these groups were excluded?**

(Our response for this comment)

We appreciate this comment. As stated in the paper, small organizations (i.e. workplaces with less than 50 employees) have different occupational healthcare requirements under Japanese law. For example, employers who hire 50 or more employees must appoint one or more occupational health physicians for advisory services.

In addition, the Stress Check Program was mandatory only for employers in larger workplaces. The data in this study contains data from workers in small workplace, despite the fact that the law does not require them to conduct the program. Possibly, such workplaces have a better understanding of mental health among workers, compared to other small workplaces which did not conduct the program. Including their data into this analysis could lower the representativeness of the Japanese population, therefore we excluded them from this study.

(Materials and methods section, page 8, line 111-116)

*In addition, workers who belong to workplaces with less than 50 employees were excluded because such workplaces have different occupational healthcare requirements under Japanese law. For example, assignment of occupational health physicians is not mandatory for such small workplaces. Furthermore, it is also not mandatory for them to conduct the Stress Check Program; therefore we excluded their data to ensure representability. Shift workers and part-time workers were excluded because their work schedule varies considerably* *different from other workers.*

***Comment #7***

**You exclude a portion of 15 per cent because of missing data. Which data were missing? For instance, if only single items or single scales were missing by these persons, wouldn’t inclusion of all available data be better? At a minimum, I would make a case that exclusion of these cases does not affect your results and conclusions. For instance, you could compare the focal (conservative) vs. the larger sample applying a more liberal approach of exclusion.**

(Our response for this comment)

We appreciate this comment. We made a table to show which variables were missing among those who have any missing (n=8560). More than 50 percent of those have missing data in “types of job”, “job-class”, “employment status” and “types of schedule”. The reason why these data are more likely to be missing is unknown. Speculatively, answer boxes for these variables were in different pages of the BJSQ, and thus a certain proportion of participants may overlooked them.

| Table. Number of missing data in each variable (n=8560) | |  |  |
| --- | --- | --- | --- |
| Variable | | Number of missing | (%) |
| Stressor | Quantitative job overload | 204 | 2% |
|  | Qualitative job overload | 167 | 2% |
|  | Physical demands | 47 | 1% |
|  | Interpersonal conflict | 200 | 2% |
|  | Poor physical environment | 104 | 1% |
|  | Job control | 118 | 1% |
|  | Skill utilization | 92 | 1% |
|  | Suitable job | 82 | 1% |
|  | Meaningfulness of work | 76 | 1% |
| Stress-response | Lack of vigor | 429 | 5% |
|  | Irritability | 385 | 4% |
|  | Fatigue | 335 | 4% |
|  | Anxiety | 466 | 5% |
|  | Depression | 544 | 6% |
|  | Somatic responses | 651 | 8% |
| Support | Support from supervisor | 468 | 5% |
|  | Support from coworker | 438 | 5% |
| Other | Length of overtime hours | 1,005 | 12% |
|  | Types of job | 5,340 | 62% |
|  | Job class | 5,576 | 65% |
|  | Employment status | 4,875 | 57% |
|  | Types of schedule | 5,218 | 61% |

In order to check whether these missing data have an impact on the result, we conducted multiple imputation procedures. And we found that these missing data did not affect the present results. These results were shown in Supplemental table 1.

| Supplemental table 2. Association between overtime working hours and stress responses : multiple linear regression results with multiple imputation | | | | | | | | | | | | | | | | | | | | | | | |
| --- | --- | --- | --- | --- | --- | --- | --- | --- | --- | --- | --- | --- | --- | --- | --- | --- | --- | --- | --- | --- | --- | --- | --- |
| Overtime working hours | Lack of Vigor | | |  | Irritability | | |  | Fatigue | | |  | Anxiety | | |  | Depression | | |  | Somatic responses | | |
|  | beta | 95％ CI | p |  | beta | 95％ CI | p |  | beta | 95％ CI | p |  | beta | 95％ CI | p |  | beta | 95％ CI | p |  | beta | 95％ CI | p |
| **Total** |  |  |  |  |  |  |  |  |  |  |  |  |  |  |  |  |  |  |  |  |  |  |  |
| ≤20 | Ref |  |  |  | Ref |  |  |  | Ref |  |  |  | Ref |  |  |  | Ref |  |  |  | Ref |  |  |
| 21-30 | 0.06 | (0.04 , 0.08) | *** |  | 0.14 | (0.12 , 0.16) | *** |  | 0.21 | (0.18 , 0.23) | *** |  | 0.15 | (0.13 , 0.17) | *** |  | 0.09 | (0.07 , 0.11) | *** |  | 0.07 | (0.04 , 0.09) | *** |
| 31-40 | 0.05 | (0.03 , 0.08) | *** |  | 0.19 | (0.16 , 0.21) | *** |  | 0.32 | (0.30 , 0.35) | *** |  | 0.23 | (0.20 , 0.25) | *** |  | 0.12 | (0.09 , 0.14) | *** |  | 0.12 | (0.09 , 0.14) | *** |
| 41-50 | 0.04 | (0.01 , 0.06) | ** |  | 0.21 | (0.18 , 0.24) | *** |  | 0.42 | (0.39 , 0.45) | *** |  | 0.28 | (0.25 , 0.31) | *** |  | 0.17 | (0.14 , 0.20) | *** |  | 0.17 | (0.14 , 0.19) | *** |
| 51-60 | -0.03 | (-0.06 , 0.00) | + |  | 0.24 | (0.20 , 0.27) | *** |  | 0.47 | (0.44 , 0.50) | *** |  | 0.32 | (0.29 , 0.35) | *** |  | 0.16 | (0.13 , 0.19) | *** |  | 0.17 | (0.14 , 0.20) | *** |
| 61-70 | -0.10 | (-0.14 , -0.06) | *** |  | 0.26 | (0.22 , 0.30) | *** |  | 0.54 | (0.50 , 0.58) | *** |  | 0.43 | (0.39 , 0.47) | *** |  | 0.22 | (0.18 , 0.26) | *** |  | 0.20 | (0.16 , 0.25) | *** |
| 71-80 | -0.12 | (-0.16 , -0.08) | *** |  | 0.35 | (0.31 , 0.40) | *** |  | 0.61 | (0.57 , 0.65) | *** |  | 0.50 | (0.46 , 0.55) | *** |  | 0.28 | (0.24 , 0.32) | *** |  | 0.27 | (0.23 , 0.31) | *** |
| 81- | 0.01 | (-0.05 , 0.07) |  |  | 0.40 | (0.34 , 0.40) | *** |  | 0.72 | (0.66 , 0.00) | *** |  | 0.46 | (0.40 , 0.51) | *** |  | 0.28 | (0.22 , 0.34) | *** |  | 0.25 | (0.19 , 0.31) | *** |
| p for linear trend | <0.001 (negative) | | |  | <0.001 (positive) | | |  | <0.001 (positive) | | |  | <0.001 (positive) | | |  | <0.001 (positive) | | |  | <0.001 (positive) | | |
| **Men** |  |  |  |  |  |  |  |  |  |  |  |  |  |  |  |  |  |  |  |  |  |  |  |
| ≤20 | Ref |  |  |  | Ref |  |  |  | Ref |  |  |  | Ref |  |  |  | Ref |  |  |  | Ref |  |  |
| 21-30 | 0.05 | (0.02 , 0.07) |  |  | 0.13 | (0.11 , 0.16) | *** |  | 0.16 | (0.14 , 0.19) | *** |  | 0.11 | (0.09 , 0.14) | *** |  | 0.05 | (0.02 , 0.07) | ** |  | 0.03 | (0.00 , 0.05) | * |
| 31-40 | 0.02 | (0.00 , 0.05) |  |  | 0.16 | (0.14 , 0.19) | *** |  | 0.27 | (0.24 , 0.29) | *** |  | 0.17 | (0.14 , 0.20) | *** |  | 0.07 | (0.04 , 0.09) | *** |  | 0.08 | (0.05 , 0.10) | *** |
| 41-50 | 0.01 | (-0.02 , 0.04) |  |  | 0.20 | (0.17 , 0.23) | *** |  | 0.37 | (0.34 , 0.40) | *** |  | 0.24 | (0.21 , 0.27) | *** |  | 0.13 | (0.10 , 0.16) | *** |  | 0.14 | (0.10 , 0.17) | *** |
| 51-60 | -0.03 | (-0.06 , 0.00) | + |  | 0.22 | (0.19 , 0.26) | *** |  | 0.43 | (0.39 , 0.46) | *** |  | 0.27 | (0.24 , 0.31) | *** |  | 0.11 | (0.08 , 0.14) | *** |  | 0.14 | (0.11 , 0.17) | *** |
| 61-70 | -0.12 | (-0.16 , -0.08) | *** |  | 0.25 | (0.20 , 0.29) | *** |  | 0.49 | (0.45 , 0.53) | *** |  | 0.38 | (0.34 , 0.42) | *** |  | 0.17 | (0.12 , 0.21) | *** |  | 0.16 | (0.11 , 0.20) | *** |
| 71-80 | -0.13 | (-0.17 , -0.08) | *** |  | 0.34 | (0.29 , 0.38) | *** |  | 0.57 | (0.52 , 0.61) | *** |  | 0.46 | (0.41 , 0.50) | *** |  | 0.23 | (0.18 , 0.27) | *** |  | 0.23 | (0.19 , 0.28) | *** |
| 81- | -0.01 | (-0.07 , 0.05) |  |  | 0.41 | (0.34 , 0.47) | *** |  | 0.72 | (0.66 , 0.78) | *** |  | 0.43 | (0.36 , 0.49) | *** |  | 0.25 | (0.19 , 0.31) | *** |  | 0.24 | (0.18 , 0.31) | *** |
| p for linear trend | <0.001 (negative) | | |  | <0.001 (positive) | | |  | <0.001 (positive) | | |  | <0.001 (positive) | | |  | <0.001 (positive) | | |  | <0.001 (positive) | | |
| **Women** |  |  |  |  |  |  |  |  |  |  |  |  |  |  |  |  |  |  |  |  |  |  |  |
| ≤20 | Ref |  |  |  | Ref |  |  |  | Ref |  |  |  | Ref |  |  |  | Ref |  |  |  | Ref |  |  |
| 21-30 | 0.08 | (0.04 , 0.12) | *** |  | 0.16 | (0.12 , 0.20) | *** |  | 0.28 | (0.24 , 0.31) | *** |  | 0.21 | (0.17 , 0.24) | *** |  | 0.16 | (0.12 , 0.19) | *** |  | 0.13 | (0.09 , 0.17) | *** |
| 31-40 | 0.15 | (0.10 , 0.20) | *** |  | 0.24 | (0.19 , 0.29) | *** |  | 0.46 | (0.41 , 0.51) | *** |  | 0.36 | (0.31 , 0.41) | *** |  | 0.21 | (0.16 , 0.26) | *** |  | 0.20 | (0.15 , 0.25) | *** |
| 41-50 | 0.14 | (0.07 , 0.21) | *** |  | 0.23 | (0.16 , 0.31) | *** |  | 0.53 | (0.46 , 0.61) | *** |  | 0.36 | (0.29 , 0.43) | *** |  | 0.24 | (0.16 , 0.31) | *** |  | 0.21 | (0.14 , 0.29) | *** |
| 51-60 | -0.03 | (-0.12 , 0.07) |  |  | 0.22 | (0.11 , 0.32) | *** |  | 0.59 | (0.49 , 0.69) | *** |  | 0.49 | (0.39 , 0.59) | *** |  | 0.34 | (0.24 , 0.43) | *** |  | 0.23 | (0.13 , 0.33) | *** |
| 61-70 | 0.11 | (-0.05 , 0.27) |  |  | 0.24 | (0.07 , 0.40) | ** |  | 0.77 | (0.61 , 0.93) | *** |  | 0.66 | (0.50 , 0.82) | *** |  | 0.54 | (0.38 , 0.69) | *** |  | 0.39 | (0.22 , 0.55) | *** |
| 71-80 | -0.11 | (-0.28 , 0.07) |  |  | 0.32 | (0.14 , 0.51) | ** |  | 0.78 | (0.60 , 0.97) | *** |  | 0.72 | (0.53 , 0.90) | *** |  | 0.60 | (0.42 , 0.77) | *** |  | 0.42 | (0.24 , 0.61) | *** |
| 81- | -0.03 | (-0.20 , 0.15) |  |  | 0.33 | (0.14 , 0.51) | *** |  | 0.57 | (0.39 , 0.76) | *** |  | 0.51 | (0.33 , 0.69) | *** |  | 0.35 | (0.17 , 0.52) | ** |  | 0.20 | (0.01 , 0.38) | * |
| p for linear trend | <0.001 (positive) | | |  | <0.001 (positive) | | |  | <0.001 (positive) | | |  | <0.001 (positive) | | |  | <0.001 (positive) | | |  | <0.001 (positive) | | |

+ p<0.1, *p<0.05, ** p<0.01, ***p<0.001, Higher score indicates unfavorable stress-response. All betas were adjusted by age, type of job, job class, employment status, type of schedule, company size, company industry, job control, supervisors support and coworker’s support. Lack of vigor was derived by reversing the score of vigor for harmonization with other stress-response scales, which higher score indicates unfavorable stress-response

***Comment #8***

**Do you have information on nesting of data (which persons belonged to which organization)? If yes, please examine the degree of nesting of data (Luke, 2004). If nesting is an issue you should take nesting into account by virtue of multilevel modeling. IF you lack information, please discuss ignorance of nesting of the data as a limitation of your study.**

(Our response for this comment)

We appreciate this important comment. Unfortunately, our data did not include detailed workplace information. Thus, we added this point in the limitation section.

(Results and discussion section: page 32, line 397-397)

*Fifth, the present study could not perform a nested data analysis due to lack of detailed workplace information. Individual stress responses could be affected not only on the individual level, but also on the group and workplace levels (59); thus future studies using multilevel analysis with detailed workplace data would be preferable (60).*

***Comment #9***

**I commend the comprehensive description of the sample**

(Our response for this comment)

We are grateful for this encouraging comment.

***Comment #10***

**Results**

**Please provide a correlation table including all focal variables. Include means, standard deviations, and a measure of reliability of multi-item scales, such as Cronbach’s Alpha or Omega. This would facilitate inclusion of your study in meta-analyses. This would also help understand the counter-intuitive results for “lack of vigor”. I would expect fatigue and vigor to be highly negatively correlated. However, in the work engagement literature there are also aspects like being very resilient while working intensively (Mills, Culbertson, & Fullagar, 2012).**

(Our response for this comment)

We appreciate this important comment. In response to this comment, we added a table (Supplemental table 1) to explain detailed BJSQ questionnaire items and Cronbach alpha for each scale. The BJSQ consists of 57 items (1 to 11 for each scale). For scales which consist of multiple items, Cronbach’s alpha ranged from 0.675 to 0.911. We added this table as a Supplemental table into the manuscript.

In addition, we checked the correlation coefficients between our stress response scales. The correlation coefficient between lack of vigor (reverse score of vigor) and fatigue was 0.341 which is moderate but in the expected direction. As stated in a previous comment, we added a table with correlations between stress response scales in Supplemental table 1-2.

***Comment #11***

**What does p for trend mean?**

(Our response for this comment)

Trend analysis aims to check the linear association between overtime working hours and stress responses by using overtime working hours as a single continuous variable in multiple linear regression. This analysis allows us to discuss whether the longer they work, the worse they become or not. To clarify this, we rephrased all “p for trend” into “p for linear trend” in tables.

***Comment #12***

**It was not totally clear to me what you did with the ANOVA. What were the factors? Were the classes of overtime hour’s one factor? Would an omnibus test really be the best approach? Given your specific expectations, a contrast analysis might be viable, too. As far as I understood Table 2, you compared all classes to the reference group of no or few hour’s overtime. A contrast analysis would help being more specific an allow comparisons between all groups.**

(Our response for this comment)

We appreciate this important comment. As we wrote in the manuscript in the first submission, we conducted analyses of covariance (ANCOVA). In the ANCOVA models, we used a score of each stress response (such as “lack of vigor” or “irritability”, etc.) as a dependent variable, “overtime work hours” as an independent variable, other personal characteristics (such as “age” and “job position”, etc.) as covariates(i.e. independent variables). In that model, “overtime work hours” were treated as dummy variables by setting “less than 20 hours” group as a reference category.

In response to this comment, we asked for statistical advice from the Biostatistician. (We acknowledged him in the manuscript). He confirmed that the current statistical analysis (i.e. ANCOVA) was not wrong, however, he suggested that this analysis should be regarded as “multiple linear regression using dummy variables”. ANCOVA would be more suitable to compare adjusted means within more “nominal variables” such as ethnicity. Therefore, we changed in all places the name of the statistical analysis from “ANCOVA” to “multiple linear regression analysis”.

(Materials and methods section, page 10-11, line 156-162)

*Multiple linear regression analyses were used to examine the associations of stress responses with overtime. In each model, we used a standardized score of each stress response (such as “lack of vigor” or “irritability”, etc.) as a dependent variable, “length of overtime work hours” as an independent variable, other individual characteristics (such as “age” and “job position”, etc.) as covariates. In that model, “length of overtime work hours” were treated as a dummy variable by setting “less than 20hours” group as a reference category. Then, linear trend tests were performed by treating “length of overtime work hours” as a continuous variable.*

(Results and discussions section, page 16-17, line 218-225)

*Table 2 shows the multiple linear regression results stratified by gender.*

We also reconsidered Figure 2 carefully. In the previous figure 2 (shown as **previous** figure below), zero value in y axis indicated the adjusted mean of whole subjects in each stress response. This adjusted mean was calculated by ANCOVA without setting any reference category. Since all stress responses were standardized prior to analysis, the adjusted mean of total subjects became zero. Whereas in other analyses such as table 2, those with 20 or less hours of overtime were used as reference category. This gap may cause confusion for readers. Therefore, to harmonize all statistical models, we decided to replace Figure 2 with the result based upon the multivariable linear regression result, setting 20 hours or less as a reference category (show as **new** figure below).

(The **pervious** Figure 2)

≤ 20	21-30	31-40	41-50	51-60	61-70	71-80	>80

Adjusted standardized scores of stress responses

|  | Lack of vigor | Irritability | Fatigue | Anxiety | Depression | Somatic responses |
| --- | --- | --- | --- | --- | --- | --- |
| 10 | -0.011259 | -0.10305 | -0.187877 | -0.138735 | -0.072727 | -0.071424 |
| 25 | 0.036639 | 0.030721 | 0.010362 | 0.00719 | -0.001085 | -0.009835 |
| 35 | 0.03149 | 0.082616 | 0.143802 | 0.095076 | 0.034532 | 0.046843 |
| 45 | 0.037901 | 0.120381 | 0.259388 | 0.177957 | 0.114598 | 0.111633 |
| 55 | 0.001174 | 0.167609 | 0.34046 | 0.248558 | 0.13438 | 0.145942 |
| 65 | -0.129117 | 0.14501 | 0.375932 | 0.325605 | 0.152438 | 0.144311 |
| 75 | -0.109451 | 0.274225 | 0.486437 | 0.425783 | 0.24253 | 0.234846 |
| 85 | 0.045011 | 0.346036 | 0.606709 | 0.383626 | 0.245299 | 0.197453 |

**Figure 2**. Adjusted means and 95% confidence intervals of standardized scores by length of overtime; analysis of covariance results

*Higher score indicates unfavorable stress-response. Adjusted covariates are gender, age, type of job, job class, employment status, type of schedule, company size, company industry, job control, supervisor’s support and coworker’s support.

(The **new** Figure 2)

≤ 20(ref)	21-30	31-40	41-50	51-60	61-70	71-80	>80

Beta for each category of overtime working hours

|  | Lack of vigor | Irritating | Fatigue | Anxiety | Depression | Somatic responses |
| --- | --- | --- | --- | --- | --- | --- |
| 10 | 0 | 0 | 0 | 0 | 0 | 0 |
| 25 | 0.055543 | 0.136615 | 0.195307 | 0.139954 | 0.0747614 | 0.0561027 |
| 35 | 0.0413707 | 0.181045 | 0.315481 | 0.212534 | 0.100571 | 0.104557 |
| 45 | 0.0235156 | 0.197713 | 0.405397 | 0.266753 | 0.153306 | 0.153725 |
| 55 | -0.0321422 | 0.226029 | 0.45918 | 0.308954 | 0.148783 | 0.168289 |
| 65 | -0.110711 | 0.245385 | 0.533993 | 0.425516 | 0.213365 | 0.19965 |
| 75 | -0.129647 | 0.344275 | 0.611847 | 0.489311 | 0.265496 | 0.261558 |
| 85 | 0.0147826 | 0.406792 | 0.731854 | 0.447715 | 0.268216 | 0.229401 |

**Figure 2**. Associations between stress response and overtime working hours; multiple linear regression results

*Higher score indicates unfavorable stress-response. Adjusted covariates are gender, age, type of job, job class, employment status, type of schedule, company size, company industry, job control, supervisor’s support and coworker’s support

**Comment #13**

Given your high number of significance tests, probably a Bonferroni-correction or something would be warranted. Given the probably very high test power, have you considered reporting effect sizes? I mean with a sample of 50,000+ almost any small effect likely will be significant.

(Our response for this comment)

We appreciate this important comment. In response to this comment, we re-calculated the same ANCOVA model with post-hoc Bonferroni correction (i.e. calculation of 99.9% confidence level) and acquired comparable results with the previous model. Therefore, we believe the results were robust.

| Table .Associations between overtime work hours and stress responses: gender-stratified analysis of multiple linear regression results (post-hoc Bonferroni correction applied) | | | | | | | | | | | |
| --- | --- | --- | --- | --- | --- | --- | --- | --- | --- | --- | --- |
| Length of overtime | Lack of Vigor | | |  | Irritability | | |  | Fatigue | | |
|  | beta | 99.9% CI | p |  | beta | 99.9% CI | p |  | beta | 99.9% CI | p |
| ≤20 | Ref |  |  |  | Ref |  |  |  | Ref |  |  |
| 21-30 | 0.06 | (0.02 , 0.09) | ** |  | 0.14 | (0.10 , 0.17) | *** |  | 0.20 | (0.16 , 0.23) | *** |
| 31-40 | 0.04 | (0.00 , 0.08) |  |  | 0.18 | (0.14 , 0.22) | *** |  | 0.32 | (0.27 , 0.36) | *** |
| 41-50 | 0.02 | (-0.02 , 0.07) |  |  | 0.20 | (0.15 , 0.25) | *** |  | 0.41 | (0.36 , 0.45) | *** |
| 51-60 | -0.03 | (-0.09 , 0.02) |  |  | 0.23 | (0.17 , 0.28) | *** |  | 0.46 | (0.40 , 0.51) | *** |
| 61-70 | -0.11 | (-0.18 , -0.04) | *** |  | 0.25 | (0.17 , 0.32) | *** |  | 0.53 | (0.46 , 0.61) | *** |
| 71-80 | -0.13 | (-0.20 , -0.06) | *** |  | 0.34 | (0.27 , 0.42) | *** |  | 0.61 | (0.54 , 0.69) | *** |
| 81- | 0.02 | (-0.09 , 0.12) |  |  | 0.41 | (0.30 , 0.51) | *** |  | 0.73 | (0.63 , 0.83) | *** |
|  | Anxiety | | |  | Depression | | |  | Somatic responses | | |
|  | beta | 99.9% CI | p |  | beta | 99.9% CI | p |  | beta | 99.9% CI | p |
| ≤20 | Ref |  |  |  | Ref |  |  |  | Ref |  |  |
| 21-30 | 0.14 | (0.10 , 0.18) | *** |  | 0.08 | (0.04 , 0.11) | ** |  | 0.06 | (0.02 , 0.09) | * |
| 31-40 | 0.21 | (0.17 , 0.25) | *** |  | 0.10 | (0.06 , 0.14) | *** |  | 0.11 | (0.06 , 0.15) | *** |
| 41-50 | 0.27 | (0.22 , 0.32) | *** |  | 0.15 | (0.10 , 0.20) | *** |  | 0.15 | (0.10 , 0.20) | *** |
| 51-60 | 0.31 | (0.25 , 0.36) | *** |  | 0.15 | (0.09 , 0.20) | *** |  | 0.17 | (0.11 , 0.22) | *** |
| 61-70 | 0.43 | (0.35 , 0.50) | *** |  | 0.21 | (0.14 , 0.29) | *** |  | 0.20 | (0.12 , 0.27) | *** |
| 71-80 | 0.49 | (0.41 , 0.56) | *** |  | 0.27 | (0.19 , 0.34) | *** |  | 0.26 | (0.18 , 0.34) | *** |
| 81- | 0.45 | (0.34 , 0.55) | *** |  | 0.27 | (0.17 , 0.37) | *** |  | 0.23 | (0.12 , 0.34) | *** |

According to Rothman’s work (Rothman KJ. No adjustments are needed for multiple comparisons, *Epidemiology*. 1990 Jan;1(1):43-6), He explained the reason as “*A policy of not making adjustments for multiple comparisons is preferable because it will lead to fewer errors of interpretation when the data under evaluation are not random numbers but actual observations on nature*.” Therefore, we have chosen not to include this result into the main manuscript.

**Comment #14**

**I think, you mention regression analysis in the methods section. What did the regression model look like? What did you analyze? Where are the results reported?**

(Our response for this comment)

We deeply apologize for this confusion. As stated in a previous comment, we decided to use the term of “multiple linear regression analysis” for the whole article.

***Comment #15***

**Discussion**

**I appreciate the comprehensive discussion of results and the acknowledgment of limitations.**

- **Demerouti, E., Mostert, K., & Bakker, A. B. (2010). Burnout and work engagement: A thorough investigation of the independency of both constructs. Journal of Occupational Health Psychology, 15(3), 209–222.** [**https://doi.org/10.1037/a0019408**](https://doi.org/10.1037/a0019408)
- **González-Romá, V., Schaufeli, W. B., Bakker, A. B., & Lloret, S. (2006). Burnout and work engagement: Independent factors or opposite poles? Journal of Vocational Behavior, 68(1), 165–174.**
- **Luke, D. A. (2004). Multilevel modeling. Thousand Oaks: Sage.**
- **Mills, M. J., Culbertson, S. S., & Fullagar, C. J. (2012). Conceptualizing and measuring engagement: An analysis of the Utrecht Work Engagement Scale. Journal of Happiness Studies, 13(3), 519–545.** [**https://doi.org/10.1007/s10902-011-9277-3**](https://doi.org/10.1007/s10902-011-9277-3)
- **Mohr, G., Müller, A., Rigotti, T., Aycan, Z., & Tschan, F. (2006). The assessment of psychological strain in work contexts: Concerning the structural equivalency of nine language adaptations of the irritation scale. European Journal of Psychological Assessment, 22(3), 198–206.** [**https://doi.org/10.1027/1015-5759.22.3.198**](https://doi.org/10.1027/1015-5759.22.3.198)

(Our response for this comment)

We appreciate this important comment. As these reference suggested, vigor is one of sub-scales for work engagement.

Vigor in work-engagement includes the concepts of “motivation” or “psychological resilience”. According to Shirom, the conceptualization of vigor is somewhat different between POMS and work-engagement. (Shirom, 2011). As stated above, the items to assess vigor in this study (i.e. BJSQ) were based on POMS.

We also think that it is important to assess how work-engagement or burnout were associated by overtime working hours, however unfortunately, our data did not include these scales.

To explain this, we added a paragraph in the manuscript.

(Results and discussion section, page 29, line 336-343)

*Vigor among workers have been gathered research attention in the context of positive occupational health psychology such as work engagement (50,51). Work engagement was defined as “a positive, fulfilling, work-related state of mind that is characterized by vigor, dedication and absorption”(52). Vigor in work-engagement is assessed by including concepts of “motivation” or “psychological resilience”, whereas the items to assess vigor in BJSQ were based on POMS. According to Shirom, the conceptualization of vigor in both work-engagement and BJSQ is not exactly the same (53). Exploring the association between overtime working hours and vigor in work engagement is an important research question in future studies.*

Reviewer #2

**General Comment #1**

**The authors present a retrospective evaluation of data from the national stress check program. In order to give feedback to participant’s data were collected in a non-anonymized manner. This needs to be specified more clearly in methods section and needs to be discussed in limitations. Subjective assessment in self-reporting measures might reveal anticipated responses and not the real situation. Most likely anticipation-bias might have influenced the responses of workers in a relationship of professional dependency.**

(Our response for this comment)

We appreciate this important comment. We agree that anticipation bias are one limitation in Stress Check Program, which might affect this results. Thus, we added this point in Method and Discussion section

(Materials and methods section, page 8, line 117-119)

*All data were collected in a non-anonymized manner to give feedback to participants in Stress Check Program, however the data was anonymized before being provided to the authors due to ethical issue.*

(Results and discussion section, page 32, line 397-400)

*Finally, the data in the present study may suffer from anticipation bias. Since participants received the Stress Check Program in non-anonymous manner, they might report their stress levels with anticipated responses, and might not sufficiently reflect the real situation.*

**General Comment #2**

**The BJSQ in its original form comprises 17 items on job stress, 29 items on psychological and physical stress reactions and 11 items on social support at work. (See: Tsutsumi A, Inoue A, Eguchi H. J Occup Health. 2017; 59(4): 356–360.)**

**Please specify whether you focused on the psychological and physical stress reactions with 29 items.**

(Our response for this comment)

We appreciate this important comment. Similar comments were also provided from another reviewer. We agree with clarifying detailed explanation with specific BJSQ items. Therefore, we added a supplemental table with citing the reference you suggested.

| Supplemental table1-1: Specific items with internal consistency of the scales of Brief Job Stress Questionnaire used in the study | | | | | | |
| --- | --- | --- | --- | --- | --- | --- |
| Category | Scale | Number of item | Question | Mean | SD | Cronbach's alpha |
| Stressor |  |  | *Please answer the following questions concerning your job by circling the number that best fits your situation. (1:Very much so, 2: Moderately so, 3: Somewhat 4: Not at all)* | - | - | - |
|  | Job control | 3 | *I can work at my own pace* | 2.64 | 0.85 | 0.712 |
|  |  |  | *I can choose how and in what order to do my work* | 2.30 | 0.80 |  |
|  |  |  | *I can reflect my opinions on workplace policy* | 2.48 | 0.79 |  |
| Stress-response | |  | *Please answer the following questions concerning your health during the past month by circling the number that best fits your situation. (1:Almost never, 2: Sometimes 3: Often 4:Almost always)* | - | - | - |
|  | Vigor | 3 | *I have been very active* | 2.15 | 0.80 | 0.911 |
|  |  |  | *I have been full of energy* | 2.21 | 0.83 |  |
|  |  |  | *I have been lively* | 2.14 | 0.82 |  |
|  | Irritability | 3 | *I have felt angry* | 2.08 | 0.81 | 0.892 |
|  |  |  | *I have been inwardly annoyed or aggravated* | 2.06 | 0.84 |  |
|  |  |  | *I have felt irritable* | 2.10 | 0.82 |  |
|  | Fatigue | 3 | *I have felt extremely tired* | 2.40 | 0.86 | 0.873 |
|  |  |  | *I have felt exhausted* | 2.04 | 0.92 |  |
|  |  |  | *I have felt weary or listless* | 2.15 | 0.89 |  |
|  | Anxiety | 3 | *I have felt tense* | 2.34 | 0.88 | 0.764 |
|  |  |  | *I have felt worried or insecure* | 2.15 | 0.93 |  |
|  |  |  | *I have felt restless* | 1.80 | 0.84 |  |
|  | Depression | 6 | *I have been depressed* | 1.93 | 0.90 | 0.885 |
|  |  |  | *I have thought that doing anything was a hassle* | 1.76 | 0.81 |  |
|  |  |  | *I have been unable to concentrate* | 1.70 | 0.73 |  |
|  |  |  | *I have felt gloomy* | 1.96 | 0.85 |  |
|  |  |  | *I have been unable to handle work* | 1.42 | 0.64 |  |
|  |  |  | *I have felt sad* | 1.47 | 0.74 |  |
|  | Somatic responses | 11 | *I have felt dizzy* | 1.34 | 0.64 | 0.841 |
|  |  |  | *I have experienced joint pains* | 1.43 | 0.73 |  |
|  |  |  | *I have experienced headaches* | 1.70 | 0.81 |  |
|  |  |  | *I have had a stiff neck and / or shoulders* | 2.41 | 1.05 |  |
|  |  |  | *I have had lower back pain* | 1.94 | 0.96 |  |
|  |  |  | *I have had eyestrain* | 2.52 | 0.95 |  |
|  |  |  | *I have experienced heart palpitations or shortness of breath* | 1.32 | 0.63 |  |
|  |  |  | *I have experienced stomach and / or intestine problems* | 1.63 | 0.81 |  |
|  |  |  | *I have lost my appetite* | 1.31 | 0.60 |  |
|  |  |  | *I have experienced diarrhea and / or constipation* | 1.74 | 0.89 |  |
|  |  |  | *I haven’t been able to sleep well* | 1.66 | 0.84 |  |
| Support |  |  | *Please answer the following questions concerning people around you by circling the number that best fits your situation. (1: Extremely, 2:Very much, 3:Somewhat, 4:Not at all)* | - | - | - |
|  | Supervisor’s support | 3 | *How freely can you talk with your supervisor?* | 2.42 | 0.81 | 0.820 |
|  |  |  | *How reliable are your supervisor when you are troubled?* | 2.30 | 0.84 |  |
|  |  |  | *How well will your supervisor listen to you when you ask for advice on personal matters?* | 2.53 | 0.87 |  |
|  | Coworker’s support | 3 | *How freely can you talk with your coworker?* | 2.07 | 0.76 | 0.793 |
|  |  |  | *How reliable are your coworker when you are troubled?* | 2.26 | 0.78 |  |
|  |  |  | *How well will your coworker listen to you when you ask for advice on personal matters?* | 2.47 | 0.82 |  |

**Specific comment #1**

**Page 1, title**

**Specify in the title that this is a retrospective cross-sectional study.**

**Suggested wording: “****Association of overtime work hours with various stress responses in 59,021 Japanese workers: retrospective cross-sectional study”**

(Our response for this comment)

We agree with showing the precise study design in the title, thus we revised the part as you suggested. We appreciate for this suggestion.

(Title, page 1, line 2-3)

*Association of overtime work hours with various stress responses in 59,021 Japanese workers: retrospective cross-sectional study*

**Specific comment #2**

**Page 3, abstract**

**Lines 35, 42…: Suggested phrasing: “****overtime work” e.g. length of overtime work**

**Page 3, abstract**

**Line 37: Suggested phrasing: “self-reporting”**

**Page 5, introduction**

**Line 58: Delete: “too much”**

(Our response for this comment)

We agree with all of these suggestions, thus we revised the part as you suggested. We appreciate for them.

**Specific comment #3**

**Page 6, study design**

**Line 81**

**To my understanding, this is a retrospective data evaluation of non-anonymized data acquired by the Stress Check Program.**

**Please specify primary and secondary study objectives**

(Our response for this comment)

We appreciate this comment. The primary objective has been stated in abstract or introduction as “*This study aims to clarify the relationships between length of overtime work and various stress responses using large-scale cross-sectional data of Japanese workers.*” Thus, in this article we describe retrospective data acquired during the conductance of the Stress Check Program.

(Materials and methods section, page 8, line 108-110)

*This research was based on data collected as part of the Stress Check Program retrospectively. The primary objective of the main project is investigating longitudinal effects of long working hours on workers´ mental and physical health.*

**Specific comment #4**

**Page 7, participants and data collection**

**Line 95:Consider phrasing: “eligible participants” …**

(Our response for this comment)

We agree with this suggestion, thus we revised the part as you suggested.

**Specific comment #5**

**Line 96:**

**December 2015 to January 2016, …**

**Provide further information on how eligible participants were contacted, e.g. did the health service company support you with epidemiologic data and mail addresses?**

**Were non-respondents repeatedly contacted?**

(Our response for this comment)

We apologize for the incorrect data collection date. The correct data collection dates were “December 2015 to November 2016 ”.

Regarding eligible participation, the law requires employers to provide all employees who are hired for regular work with the opportunity to participate in the Stress Check Program. Based on the information provided by the company, the health service company issues IDs for all employees. We defined employees who have their own ID as eligible participants.

Since the data had been anonymized (dropping any individual mail addresses) at the health service company prior to analyses, we could not reach individual workers.

Regarding reminders to non-responders, we unfortunately do not have any detailed information of reminding. However, according to the protocol issued by Ministry of Health, Labor and Welfare, employers are encouraged to remind the Stress Check Program to non-responders. Therefore, non-responders have been reminded at least one more time. We added this information in Materials and methods section.

（Figure 1）

*Total number of regular workers at workplaces which were offered legally based Stress Check Program from December 2015 to November 2016.*

(Materials and methods section, Page 7, Line 91-98)

*The program requires all workplaces with 50 or more employees to conduct a questionnaire survey regarding psychosocial stress at work at least once a year. Feedback is provided to each employee with aims to decrease the risk of mental health problems through enhancing their awareness of their own stress (26). The law requires employers to provide all regularly hired employees with opportunities to participate in the Stress Check Program.* *According to the protocol issued by the Japanese government, employers are encouraged to remind non-responders to complete the Stress Check Program at least one time (25).*

**Specific comment #6**

**Page 8, stress responses**

**Line 119: Please specify whether you focused on the psychological and physical stress reactions with 29 items.**

**It would be helpful to the reader to know the original wording of the 29 items. Please enclose the questionnaire as appendix or as supplementary information to this manuscript.**

(Our response for this comment)

We appreciate this comment. Another reviewer also requires original wording of BJSQ with Cronbach’s alpha for each scale. Thus, we added this information in Supplemental table 1.

**Specific comment #7**

Page 9, covariates

Line 131: Does history of diseases mean past medical history? This would mean a detailed register of previous diseases and provide the base for correlations between general health and number of overtime hours per month.

(Our response for this comment)

We deeply apologize this mistake. As stated in limitation, our data did not include any data regarding past history. We deleted this from the manuscript.

**Specific comment #8**

**Page 9, statistical procedure**

**Line 140: The authors used multiple linear regression fitting a linear equation to independent variables and associated dependent variables. Multiple imputation was used for missing data management.**

**As there is known confounding by gender, qualification, leading position and age group I suggest to evaluate subgroups male/female in leading/non-leading position and age groups, in particular <30, 31-59, and >60 years (the range was 17 to 89 years).**

**Ordinal variables could be analyzed with the Mann-Whitney U test (n=2) and the Kruskal-Wallis test (n>2) in subgroup analysis.**

(Our response for this comment)

We appreciate this comment. In response to this comment, we added two stratified analyses, one is with age group and the other is with leading/non leading position. Both analyses were conducted separately by gender. We believe these results have not been covered sufficiently in past studies, therefore we added them into the main results as table 3-4.

**Specific comment #9**

**Page 11, results and discussions**

**Line 164: Suggested wording: “did not receive” …**

**Line 170: Please distinguish between response rate and inclusion rate.**

**Out of 95,004 eligible workers, 88.988 were contacted of whom 83,470 responded (response rate: 87.9%). After exclusion of 24,449 workers, a total of 59,021 participants were included (inclusion rate: 62.1%)**

(Our response for this comment)

We agree with all these suggestions, thus we revised the part as you suggested.

**Specific comment #10**

**Page 12, Table 1**

**It should be mentioned in results that the** **core group comprised** **middle-aged, male clerk with regular employment status and inflexible type of schedule. Consider that majority of workers reported overtime work of less than 20 hours a month.**

(Our response for this comment)

We appreciate this comment. In response to this comment, we added a short description of study participants in the result section.

(Results and discussions section, page 13, line 193-195)

*Table 1 shows the characteristics of study participants. The mean (±standard deviation) age was 44.3 (±10.7) years. Men accounted for 69.1 % of the participants. The core group comprised middle-aged, male clerks with regular employment status and inflexible type of schedule.*

**Specific comment #11**

**Page 14**

**Results of questionnaires are based on subjective assessments. Better write “somebody reported” instead of “somebody was”.**

(Our response for this comment)

We agree with all these suggestions, thus we revised the part as you suggested.

(Results and discussions section, page 16-17, line 218-225)

*Table 2 shows the multiple linear regression results stratified by gender. For both genders, workers with 21 hours/month or more overtime reported significantly higher “irritability”, “fatigue”, “anxiety”, “depression” and “somatic responses”, compared to those with less than 20 hours/month of overtime (p<0.001). In addition, workers who engaged in 21-30 hours/month of overtime reported a higher level of “lack of vigor”. However, male workers who engaged in 61-80 hours/month of overtime reported a significantly lower level of “lack of vigor” than those with less than 20 hours/month of overtime (51-60 hours/month: beta=-0.04 [95%CI: -0.07, 0.00], 61-70 hours/month: beta=-0.13 [-0.17. -0.09], 71-80 hours/month: beta= -0.13 [-0.18, -0.09])*

**Specific comment #12**

**A subgroup evaluation of age groups, in particular <30, 31-59, and >60 years might reveal striking differences in perceived fatigue and lack of vigor.**

(Our response for this comment)

We appreciate this important suggestion for additional analysis. In response to this comment, we added a stratified analysis by age-group. The main results were not significantly different from the results with all samples, however, this is very informative. Therefore, we added these results in the manuscript.

(Materials and methods section, page 11, line 168-171)

*Finally, stratified analyses by gender, age-category (i.e. less than 30, 30-59 and 60 or older) and job-position (regular and manager) were performed. In these models, interaction terms between age-category/job-position and overtime working hours were used to consider effect modification by gender, age and job-position.*

(Results and discussions section, page 18, line 240-246)

*Table 3 and 4 showed stratified analysis results by age-category and job-class. Compared to middle-aged male workers, older workers showed relatively higher stress responses, especially fatigue, anxiety and depression. This may suggest that older workers constitute a more vulnerable population than younger ones for overtime working than middle-aged workers. Due to the small number of working women in higher job-positions, the interaction by job-class was not clear. It is interesting that the highest beta coefficient in point was observed for fatigue among women in administrative positions (beta=1.16 [0.68. 1.63]).*

(Added tables)

Table 3: Association between overtime work hours and stress responses: results of multiple linear regression stratified by gender and age-category

| **Table 3** . Association between overtime work hours and stress responses: results of multiple linear regression stratified by gender and age-category | | | | | | | | | | | | | | | | | | | | | | | | |
| --- | --- | --- | --- | --- | --- | --- | --- | --- | --- | --- | --- | --- | --- | --- | --- | --- | --- | --- | --- | --- | --- | --- | --- | --- |
| Overtime work  hours/month | n | Lack of Vigor | | |  | Irritability | | |  | Fatigue | | |  | Anxiety | | |  | Depression | | |  | Somatic responses | | |
|  |  | beta | 95％ CI | p |  | beta | 95％ CI | p |  | beta | 95％ CI | p |  | beta | 95％ CI | p |  | beta | 95％ CI | p |  | beta | 95％ CI | p |
| **Men** |  |  |  |  |  |  |  |  |  |  |  |  |  |  |  |  |  |  |  |  |  |  |  |  |
| **Younger (aged less than 30 years)** | | | |  |  |  |  |  |  |  |  |  |  |  |  |  |  |  |  |  |  |  |  |  |
| ≤20 | 1,485 | Ref |  |  |  | Ref |  |  |  | Ref |  |  |  | Ref |  |  |  | Ref |  |  |  | Ref |  |  |
| 21-30 | 796 | 0.12 | (0.04 , 0.20) | ** |  | 0.15 | (0.07 , 0.24) | *** |  | 0.22 | (0.14 , 0.30) | *** |  | 0.12 | (0.03 , 0.20) | ** |  | 0.12 | (0.04 , 0.21) | ** |  | 0.03 | (-0.06 , 0.12) |  |
| 31-40 | 546 | 0.08 | (-0.01 , 0.17) | + |  | 0.17 | (0.07 , 0.27) | ** |  | 0.28 | (0.19 , 0.37) | *** |  | 0.15 | (0.05 , 0.24) | ** |  | 0.06 | (-0.03 , 0.16) |  |  | 0.10 | (0.00 , 0.20) |  |
| 41-50 | 345 | 0.08 | (-0.03 , 0.18) |  |  | 0.21 | (0.09 , 0.33) | ** |  | 0.45 | (0.35 , 0.56) | *** |  | 0.30 | (0.19 , 0.42) | *** |  | 0.28 | (0.17 , 0.40) | *** |  | 0.32 | (0.20 , 0.44) | *** |
| 51-60 | 291 | -0.05 | (-0.17 , 0.07) |  |  | 0.29 | (0.16 , 0.42) | *** |  | 0.48 | (0.36 , 0.59) | *** |  | 0.28 | (0.15 , 0.41) | *** |  | 0.14 | (0.01 , 0.26) | * |  | 0.15 | (0.01 , 0.28) | * |
| 61-70 | 146 | -0.01 | (-0.16 , 0.14) |  |  | 0.35 | (0.18 , 0.52) | *** |  | 0.57 | (0.42 , 0.72) | *** |  | 0.49 | (0.33 , 0.66) | *** |  | 0.37 | (0.20 , 0.54) | ** |  | 0.30 | (0.13 , 0.48) | *** |
| 71-80 | 135 | -0.12 | (-0.28 , 0.04) |  |  | 0.40 | (0.22 , 0.58) | *** |  | 0.42 | (0.26 , 0.58) | *** |  | 0.51 | (0.34 , 0.69) | *** |  | 0.41 | (0.23 , 0.59) | *** |  | 0.38 | (0.20 , 0.56) | *** |
| 81- | 77 | 0.05 | (-0.16 , 0.25) |  |  | 0.48 | (0.26 , 0.71) | *** |  | 0.89 | (0.69 , 1.09) | *** |  | 0.44 | (0.22 , 0.66) | *** |  | 0.44 | (0.22 , 0.66) | ** |  | 0.27 | (0.04 , 0.50) | * |
| p for linear trend | | <0.001 (negative) | | |  | <0.001 (positive) | | |  | <0.001 (positive) | | |  | <0.001 (positive) | | |  | <0.001 (positive) | | |  | <0.001 (positive) | | |
| **Middle (aged 30-59 years)** | | |  |  |  |  |  |  |  |  |  |  |  |  |  |  |  |  |  |  |  |  |  |  |
| ≤20 | 12,097 | Ref |  |  |  | Ref |  |  |  | Ref |  |  |  | Ref |  |  |  | Ref |  |  |  | Ref |  |  |
| 21-30 | 6,132 | 0.01 | (-0.02 , 0.03) |  |  | 0.11 | (0.08 , 0.14) | *** |  | 0.13 | (0.10 , 0.16) | *** |  | 0.08 | (0.05 , 0.11) | *** |  | 0.01 | (-0.02 , 0.04) |  |  | 0.01 | (-0.02 , 0.04) |  |
| 31-40 | 5,038 | -0.03 | (-0.06 , 0.00) | * |  | 0.14 | (0.11 , 0.17) | *** |  | 0.24 | (0.21 , 0.27) | *** |  | 0.13 | (0.10 , 0.16) | *** |  | 0.03 | (0.00 , 0.06) | + |  | 0.04 | (0.01 , 0.07) | ** |
| 41-50 | 3,624 | -0.04 | (-0.07 , 0.00) | * |  | 0.16 | (0.13 , 0.20) | *** |  | 0.33 | (0.30 , 0.36) | *** |  | 0.19 | (0.15 , 0.22) | *** |  | 0.08 | (0.04 , 0.11) | *** |  | 0.09 | (0.06 , 0.13) | *** |
| 51-60 | 3,129 | -0.07 | (-0.10 , -0.03) | *** |  | 0.19 | (0.15 , 0.22) | *** |  | 0.39 | (0.35 , 0.42) | *** |  | 0.22 | (0.19 , 0.26) | *** |  | 0.07 | (0.04 , 0.11) | *** |  | 0.11 | (0.08 , 0.15) | *** |
| 61-70 | 1,617 | -0.17 | (-0.21 , -0.12) | *** |  | 0.20 | (0.16 , 0.25) | *** |  | 0.45 | (0.41 , 0.50) | *** |  | 0.33 | (0.28 , 0.37) | *** |  | 0.12 | (0.07 , 0.17) | *** |  | 0.13 | (0.08 , 0.18) | *** |
| 71-80 | 1,577 | -0.17 | (-0.22 , -0.12) | *** |  | 0.30 | (0.25 , 0.35) | *** |  | 0.56 | (0.51 , 0.61) | *** |  | 0.40 | (0.35 , 0.45) | *** |  | 0.18 | (0.13 , 0.22) | *** |  | 0.19 | (0.14 , 0.24) | *** |
| 81- | 683 | -0.04 | (-0.10 , 0.03) |  |  | 0.40 | (0.33 , 0.47) | *** |  | 0.70 | (0.63 , 0.77) | *** |  | 0.40 | (0.33 , 0.47) | *** |  | 0.20 | (0.13 , 0.27) | *** |  | 0.20 | (0.13 , 0.27) | *** |
| p for linear trend | | <0.001 (negative) | | |  | <0.001 (positive) | | |  | <0.001 (positive) | | |  | <0.001 (positive) | | |  | <0.001 (positive) | | |  | <0.001 (positive) | | |
| **Older (aged 60 or older)** | | |  |  |  |  |  |  |  |  |  |  |  |  |  |  |  |  |  |  |  |  |  |  |
| ≤20 | 2,169 | Ref |  |  |  | Ref |  |  |  | Ref |  |  |  | Ref |  |  |  | Ref |  |  |  | Ref |  |  |
| 21-30 | 332 | 0.01 | (-0.09 , 0.12) |  |  | 0.08 | (-0.02 , 0.18) |  |  | 0.24 | (0.15 , 0.33) | *** |  | 0.23 | (0.13 , 0.32) | *** |  | 0.13 | (0.03 , 0.22) | ** |  | 0.07 | (-0.04 , 0.17) |  |
| 31-40 | 228 | 0.09 | (-0.03 , 0.22) |  |  | 0.29 | (0.17 , 0.41) | *** |  | 0.34 | (0.23 , 0.45) | *** |  | 0.31 | (0.20 , 0.42) | *** |  | 0.23 | (0.12 , 0.34) | *** |  | 0.22 | (0.09 , 0.34) | ** |
| 41-50 | 128 | -0.06 | (-0.22 , 0.10) |  |  | 0.26 | (0.10 , 0.41) | ** |  | 0.52 | (0.37 , 0.66) | *** |  | 0.43 | (0.28 , 0.57) | *** |  | 0.30 | (0.16 , 0.45) | *** |  | 0.30 | (0.13 , 0.46) | *** |
| 51-60 | 74 | -0.04 | (-0.25 , 0.17) |  |  | 0.27 | (0.06 , 0.46) | * |  | 0.57 | (0.38 , 0.75) | *** |  | 0.48 | (0.29 , 0.66) | *** |  | 0.30 | (0.11 , 0.48) | ** |  | 0.37 | (0.16 , 0.58) | ** |
| 61-70 | 51 | -0.36 | (-0.61 , -0.11) | ** |  | 0.15 | (-0.09 , 0.39) |  |  | 0.50 | (0.28 , 0.72) | *** |  | 0.51 | (0.29 , 0.73) | *** |  | 0.20 | (-0.02 , 0.42) | + |  | 0.20 | (-0.05 , 0.45) |  |
| 71-80 | 28 | 0.14 | (-0.20 , 0.47) |  |  | 0.40 | (0.08 , 0.72) | * |  | 0.68 | (0.38 , 0.97) | *** |  | 0.73 | (0.44 , 1.03) | *** |  | 0.31 | (0.02 , 0.61) | * |  | 0.47 | (0.14 , 0.81) | ** |
| 81- | 36 | 0.05 | (-0.25 , 0.35) |  |  | 0.10 | (-0.18 , 0.38) |  |  | 0.59 | (0.33 , 0.85) | *** |  | 0.32 | (0.06 , 0.58) | ** |  | 0.16 | (-0.11 , 0.42) |  |  | 0.29 | (-0.01 , 0.58) | + |
| p for linear trend | | n.s. | | |  | <0.001 (positive) | | |  | <0.001 (positive) | | |  | <0.001 (positive) | | |  | <0.001 (positive) | | |  | <0.001 (positive) | | |
| **Interaction between overtime and age-category** | | | | |  |  |  |  |  |  |  |  |  |  |  |  |  |  |  |  |  |  |  |  |
| **Middle (ref) versus Young** | | |  |  |  |  |  |  |  |  |  |  |  |  |  |  |  |  |  |  |  |  |  |  |
| 21-30 |  | 0.14 | (0.05 , 0.21) | ** |  | 0.04 | (-0.04 , 0.13) |  |  | 0.06 | (-0.02 , 0.15) |  |  | 0.03 | (-0.05 , 0.11) |  |  | 0.11 | (0.03 , 0.19) | * |  | 0.04 | (-0.05 , 0.13) |  |
| 31-40 |  | 0.16 | (0.07 , 0.25) | ** |  | 0.04 | (-0.06 , 0.13) |  |  | 0.03 | (-0.07 , 0.12) |  |  | 0.01 | (-0.08 , 0.10) |  |  | 0.04 | (-0.06 , 0.13) |  |  | 0.08 | (-0.02 , 0.17) |  |
| 41-50 |  | 0.16 | (0.05 , 0.27) | ** |  | 0.06 | (-0.05 , 0.17) |  |  | 0.10 | (-0.01 , 0.21) |  |  | 0.10 | (-0.02 , 0.21) |  |  | 0.21 | (0.10 , 0.32) | *** |  | 0.24 | (0.13 , 0.36) | *** |
| 51-60 |  | 0.09 | (-0.02 , 0.21) |  |  | 0.12 | (0.00 , 0.24) | * |  | 0.07 | (-0.05 , 0.18) |  |  | 0.04 | (-0.08 , 0.15) |  |  | 0.08 | (-0.04 , 0.20) |  |  | 0.06 | (-0.06 , 0.18) |  |
| 61-70 |  | 0.24 | (0.08 , 0.39) | ** |  | 0.18 | (0.01 , 0.34) | * |  | 0.13 | (-0.03 , 0.29) |  |  | 0.17 | (0.01 , 0.33) | * |  | 0.30 | (0.14 , 0.46) | *** |  | 0.20 | (0.03 , 0.37) | * |
| 71-80 |  | 0.13 | (-0.03 , 0.29) |  |  | 0.12 | (-0.05 , 0.28) |  |  | -0.14 | (-0.30 , 0.02) | + |  | 0.11 | (-0.06 , 0.27) |  |  | 0.27 | (0.10 , 0.44) | ** |  | 0.22 | (0.05 , 0.39) | * |
| 81- |  | 0.08 | (-0.13 , 0.29) |  |  | 0.08 | (-0.14 , 0.30) |  |  | 0.14 | (-0.07 , 0.36) |  |  | 0.01 | (-0.21 , 0.22) |  |  | 0.19 | (-0.03 , 0.41) | + |  | 0.06 | (-0.16 , 0.29) |  |
| **Middle (ref) versus Older** | | |  |  |  |  |  |  |  |  |  |  |  |  |  |  |  |  |  |  |  |  |  |  |
| 21-30 |  | 0.06 | (-0.04 , 0.17) |  |  | -0.01 | (-0.12 , 0.10) |  |  | 0.12 | (0.02 , 0.23) | * |  | 0.16 | (0.05 , 0.27) | ** |  | 0.14 | (0.03 , 0.24) | * |  | 0.10 | (-0.01 , 0.21) | + |
| 31-40 |  | 0.21 | (0.09 , 0.33) | ** |  | 0.20 | (0.07 , 0.33) | * |  | 0.14 | (0.01 , 0.26) | * |  | 0.19 | (0.07 , 0.32) | ** |  | 0.22 | (0.10 , 0.35) | ** |  | 0.23 | (0.10 , 0.36) | *** |
| 41-50 |  | 0.09 | (-0.07 , 0.25) |  |  | 0.17 | (0.00 , 0.33) | + |  | 0.25 | (0.09 , 0.41) | * |  | 0.25 | (0.09 , 0.42) | ** |  | 0.27 | (0.10 , 0.43) | ** |  | 0.27 | (0.10 , 0.44) | ** |
| 51-60 |  | 0.15 | (-0.05 , 0.36) |  |  | 0.16 | (-0.05 , 0.38) |  |  | 0.24 | (0.03 , 0.45) | * |  | 0.25 | (0.04 , 0.46) | * |  | 0.27 | (0.05 , 0.48) | * |  | 0.33 | (0.11 , 0.55) | ** |
| 61-70 |  | -0.08 | (-0.33 , 0.17) |  |  | 0.04 | (-0.22 , 0.29) |  |  | 0.12 | (-0.13 , 0.38) |  |  | 0.20 | (-0.06 , 0.45) |  |  | 0.14 | (-0.12 , 0.40) |  |  | 0.14 | (-0.12 , 0.41) |  |
| 71-80 |  | 0.40 | (0.07 , 0.73) | * |  | 0.18 | (-0.16 , 0.53) |  |  | 0.18 | (-0.15 , 0.51) |  |  | 0.35 | (0.01 , 0.69) | * |  | 0.21 | (-0.13 , 0.55) |  |  | 0.36 | (0.01 , 0.72) | * |
| 81- |  | 0.09 | (-0.20 , 0.39) |  |  | -0.30 | (-0.61 , 0.01) | + |  | -0.15 | (-0.45 , 0.16) |  |  | -0.06 | (-0.36 , 0.25) |  |  | -0.02 | (-0.32 , 0.29) |  |  | 0.08 | (-0.23 , 0.40) |  |
| **Women** |  |  |  |  |  |  |  |  |  |  |  |  |  |  |  |  |  |  |  |  |  |  |  |  |
| **Younger (aged less than 30 years)** | | | |  |  |  |  |  |  |  |  |  |  |  |  |  |  |  |  |  |  |  |  |  |
| ≤20 | 1,665 | Ref |  |  |  | Ref |  |  |  | Ref |  |  |  | Ref |  |  |  | Ref |  |  |  | Ref |  |  |
| 21-30 | 473 | 0.02 | (-0.07 , 0.11) |  |  | 0.13 | (0.02 , 0.23) | * |  | 0.23 | (0.14 , 0.33) | *** |  | 0.09 | (-0.02 , 0.19) | + |  | 0.10 | (0.00 , 0.20) | * |  | 0.08 | (-0.02 , 0.18) |  |
| 31-40 | 246 | 0.14 | (0.01 , 0.26) | * |  | 0.18 | (0.04 , 0.32) | * |  | 0.37 | (0.24 , 0.49) | *** |  | 0.25 | (0.12 , 0.38) | *** |  | 0.26 | (0.13 , 0.39) | *** |  | 0.06 | (-0.08 , 0.20) |  |
| 41-50 | 96 | 0.11 | (-0.08 , 0.29) |  |  | 0.10 | (-0.11 , 0.31) |  |  | 0.46 | (0.26 , 0.65) | *** |  | 0.34 | (0.13 , 0.54) | ** |  | 0.23 | (0.03 , 0.43) | * |  | 0.09 | (-0.12 , 0.30) |  |
| 51-60 | 81 | 0.11 | (-0.09 , 0.32) |  |  | 0.11 | (-0.12 , 0.34) |  |  | 0.66 | (0.44 , 0.87) | *** |  | 0.47 | (0.25 , 0.69) | *** |  | 0.47 | (0.25 , 0.69) | *** |  | 0.24 | (0.01 , 0.47) | * |
| 61-70 | 15 | 0.45 | (-0.01 , 0.91) |  |  | 0.26 | (-0.26 , 0.79) |  |  | 0.58 | (0.10 , 1.06) | * |  | 0.22 | (-0.28 , 0.72) |  |  | 0.49 | (0.00 , 0.98) | + |  | 0.31 | (-0.21 , 0.83) |  |
| 71-80 | 16 | -0.24 | (-0.67 , 0.20) |  |  | 0.53 | (0.02 , 1.03) | * |  | 0.78 | (0.32 , 1.25) | ** |  | 0.73 | (0.24 , 1.21) | ** |  | 0.69 | (0.22 , 1.16) | ** |  | 0.35 | (-0.15 , 0.85) |  |
| 81- | 20 | -0.06 | (-0.46 , 0.33) |  |  | 0.06 | (-0.39 , 0.51) |  |  | 0.54 | (0.12 , 0.95) | * |  | 0.24 | (-0.20 , 0.67) |  |  | 0.37 | (-0.05 , 0.80) | + |  | 0.06 | (-0.38 , 0.51) |  |
| p for linear trend | | <0.001 (positive) | | |  | <0.001 (positive) | | |  | <0.001 (positive) | | |  | <0.001 (positive) | | |  | <0.001 (positive) | | |  | <0.001 (positive) | | |
| **Middle (aged 30-59 years)** | |  | | |  |  | | |  |  | | |  |  | | |  |  | | |  |  | | |
| ≤20 | 10,896 | Ref |  |  |  | Ref |  |  |  | Ref |  |  |  | Ref |  |  |  | Ref |  |  |  | Ref |  |  |
| 21-30 | 1,735 | 0.08 | (0.03 , 0.13) | ** |  | 0.15 | (0.10 , 0.20) | *** |  | 0.27 | (0.22 , 0.32) | *** |  | 0.20 | (0.15 , 0.25) | *** |  | 0.13 | (0.08 , 0.17) | *** |  | 0.12 | (0.07 , 0.17) | *** |
| 31-40 | 942 | 0.16 | (0.10 , 0.22) | *** |  | 0.24 | (0.18 , 0.30) | *** |  | 0.50 | (0.44 , 0.57) | *** |  | 0.39 | (0.32 , 0.45) | *** |  | 0.19 | (0.12 , 0.25) | *** |  | 0.22 | (0.16 , 0.29) | *** |
| 41-50 | 462 | 0.13 | (0.05 , 0.22) | ** |  | 0.22 | (0.12 , 0.30) | *** |  | 0.53 | (0.44 , 0.62) | *** |  | 0.33 | (0.24 , 0.42) | *** |  | 0.21 | (0.13 , 0.29) | *** |  | 0.18 | (0.09 , 0.27) | *** |
| 51-60 | 240 | -0.12 | (-0.23 , 0.00) | + |  | 0.23 | (0.10 , 0.35) | *** |  | 0.57 | (0.44 , 0.70) | *** |  | 0.49 | (0.37 , 0.62) | *** |  | 0.26 | (0.14 , 0.38) | *** |  | 0.24 | (0.12 , 0.37) | *** |
| 61-70 | 110 | 0.05 | (-0.13 , 0.22) |  |  | 0.22 | (0.04 , 0.40) | * |  | 0.84 | (0.65 , 1.02) | *** |  | 0.77 | (0.59 , 0.95) | *** |  | 0.54 | (0.37 , 0.71) | *** |  | 0.41 | (0.23 , 0.59) | *** |
| 71-80 | 81 | -0.13 | (-0.33 , 0.07) |  |  | 0.34 | (0.13 , 0.55) | ** |  | 0.77 | (0.56 , 0.98) | *** |  | 0.68 | (0.47 , 0.89) | *** |  | 0.56 | (0.36 , 0.76) | *** |  | 0.44 | (0.23 , 0.65) | *** |
| 81- | 69 | 0.05 | (-0.17 , 0.26) |  |  | 0.41 | (0.18 , 0.64) | *** |  | 0.64 | (0.41 , 0.87) | *** |  | 0.61 | (0.38 , 0.84) | ** |  | 0.34 | (0.13 , 0.56) | ** |  | 0.13 | (-0.10 , 0.36) |  |
| p for linear trend | | <0.001 (positive) | | |  | <0.001 (positive) | | |  | <0.001 (positive) | | |  | <0.001 (positive) | | |  | <0.001 (positive) | | |  | <0.001 (positive) | | |
| **Older (aged 60 or older)** | | |  |  |  |  |  |  |  |  |  |  |  |  |  |  |  |  |  |  |  |  |  |  |
| ≤20 | 968 | Ref |  |  |  | Ref |  |  |  | Ref |  |  |  | Ref |  |  |  | Ref |  |  |  | Ref |  |  |
| 21-30 | 93 | 0.17 | (-0.04 , 0.39) |  |  | 0.22 | (0.03 , 0.41) | * |  | 0.27 | (0.08 , 0.46) | ** |  | 0.29 | (0.11 , 0.47) | ** |  | 0.23 | (0.06 , 0.39) | ** |  | 0.08 | (-0.12 , 0.28) |  |
| 31-40 | 31 | -0.16 | (-0.52 , 0.20) |  |  | 0.21 | (-0.11 , 0.53) |  |  | 0.23 | (-0.09 , 0.54) |  |  | 0.21 | (-0.09 , 0.51) |  |  | 0.08 | (-0.20 , 0.36) |  |  | 0.09 | (-0.24 , 0.41) |  |
| 41-50 | 9 | 0.09 | (-0.56 , 0.75) |  |  | 0.35 | (-0.24 , 0.93) |  |  | 0.31 | (-0.27 , 0.89) |  |  | 0.67 | (0.12 , 1.21) | * |  | 0.45 | (-0.06 , 0.97) | + |  | -0.02 | (-0.62 , 0.58) |  |
| 51-60 | 5 | 0.99 | (0.12 , 1.87) | * |  | 1.41 | (0.63 , 2.19) | *** |  | 1.02 | (0.24 , 1.80) | * |  | 0.71 | (-0.02 , 1.44) | + |  | 1.08 | (0.39 , 1.77) | ** |  | 0.35 | (-0.45 , 1.16) |  |
| 61-70 | 2 | 1.36 | (-0.02 , 2.74) | + |  | 1.10 | (-0.13 , 2.33) | + |  | 1.16 | (-0.07 , 2.38) |  |  | 0.56 | (-0.60 , 1.71) |  |  | 0.51 | (-0.58 , 1.60) |  |  | 0.21 | (-1.06 , 1.48) |  |
| 71-80 | 0 | - |  |  |  | - |  |  |  | - |  |  |  | - |  |  |  | - |  |  |  | - |  |  |
| 81- | 2 | -0.23 | (-1.61 , 1.16) |  |  | -0.37 | (-1.61 , 0.86) |  |  | 0.82 | (-0.41 , 2.05) |  |  | -0.18 | (-1.34 , 0.98) |  |  | -0.66 | (-1.75 , 0.44) |  |  | 0.32 | (-0.96 , 1.60) |  |
| p for linear trend | | n.s. | | |  | <0.001 (positive) | | |  | <0.001 (positive) | | |  | <0.001 (positive) | | |  | <0.01 (positive) | | |  | n.s. | | |
| **Interaction between overtime and age-category** | | | | |  |  |  |  |  |  |  |  |  |  |  |  |  |  |  |  |  |  |  |  |
| **Middle (ref) versus Young** | | |  |  |  |  |  |  |  |  |  |  |  |  |  |  |  |  |  |  |  |  |  |  |
| 21-30 |  | -0.05 | (-0.15 , 0.06) |  |  | -0.02 | (-0.13 , 0.09) |  |  | -0.05 | (-0.16 , 0.07) |  |  | -0.14 | (-0.25 , -0.03) | * |  | -0.03 | (-0.13 , 0.07) |  |  | -0.03 | (-0.14 , 0.08) | * |
| 31-40 |  | -0.01 | (-0.15 , 0.12) |  |  | -0.08 | (-0.23 , 0.06) |  |  | -0.15 | (-0.29 , 0.00) | * |  | -0.16 | (-0.30 , -0.01) | * |  | 0.06 | (-0.08 , 0.20) |  |  | -0.15 | (-0.29 , 0.00) |  |
| 41-50 |  | 0.00 | (-0.20 , 0.21) |  |  | -0.14 | (-0.36 , 0.08) |  |  | -0.07 | (-0.29 , 0.15) |  |  | -0.02 | (-0.24 , 0.20) |  |  | 0.01 | (-0.20 , 0.21) |  |  | -0.06 | (-0.28 , 0.16) |  |
| 51-60 |  | 0.27 | (0.04 , 0.51) | * |  | -0.15 | (-0.40 , 0.10) |  |  | 0.09 | (-0.16 , 0.34) |  |  | -0.06 | (-0.30 , 0.19) |  |  | 0.20 | (-0.03 , 0.44) | + |  | 0.05 | (-0.20 , 0.29) |  |
| 61-70 |  | 0.39 | (-0.11 , 0.89) |  |  | 0.00 | (-0.52 , 0.53) |  |  | -0.24 | (-0.76 , 0.29) |  |  | -0.54 | (-1.06 , -0.03) | * |  | -0.11 | (-0.60 , 0.39) |  |  | -0.05 | (-0.58 , 0.47) |  |
| 71-80 |  | -0.07 | (-0.56 , 0.42) |  |  | 0.18 | (-0.34 , 0.70) |  |  | 0.06 | (-0.47 , 0.58) |  |  | 0.09 | (-0.42 , 0.61) |  |  | 0.18 | (-0.31 , 0.67) |  |  | -0.04 | (-0.56 , 0.49) |  |
| 81- |  | -0.12 | (-0.58 , 0.34) |  |  | -0.35 | (-0.84 , 0.13) |  |  | -0.09 | (-0.58 , 0.39) |  |  | -0.35 | (-0.83 , 0.12) |  |  | 0.03 | (-0.43 , 0.48) |  |  | -0.04 | (-0.52 , 0.45) |  |
| **Middle (ref) versus Older** | | |  |  |  |  |  |  |  |  |  |  |  |  |  |  |  |  |  |  |  |  |  |  |
| 21-30 |  | 0.12 | (-0.08 , 0.32) |  |  | 0.10 | (-0.11 , 0.31) |  |  | 0.06 | (-0.15 , 0.27) |  |  | 0.13 | (-0.08 , 0.33) |  |  | 0.14 | (-0.06 , 0.33) |  |  | 0.01 | (-0.20 , 0.22) |  |
| 31-40 |  | -0.27 | (-0.60 , 0.06) |  |  | 0.01 | (-0.35 , 0.36) |  |  | -0.20 | (-0.55 , 0.16) |  |  | -0.14 | (-0.48 , 0.21) |  |  | -0.08 | (-0.41 , 0.25) |  |  | -0.07 | (-0.42 , 0.29) |  |
| 41-50 |  | 0.02 | (-0.59 , 0.63) |  |  | 0.17 | (-0.48 , 0.81) |  |  | -0.14 | (-0.78 , 0.50) |  |  | 0.34 | (-0.29 , 0.97) |  |  | 0.26 | (-0.35 , 0.86) |  |  | -0.12 | (-0.76 , 0.52) |  |
| 51-60 |  | 1.28 | (0.47 , 2.10) | ** |  | 1.32 | (0.46 , 2.18) | ** |  | 0.65 | (-0.21 , 1.50) |  |  | 0.31 | (-0.53 , 1.16) |  |  | 0.89 | (0.08 , 1.70) | * |  | 0.28 | (-0.58 , 1.14) |  |
| 61-70 |  | 1.33 | (0.04 , 2.61) | * |  | 0.82 | (-0.54 , 2.18) |  |  | 0.34 | (-1.01 , 1.69) |  |  | -0.25 | (-1.59 , 1.08) |  |  | -0.01 | (-1.28 , 1.26) |  |  | -0.13 | (-1.48 , 1.23) |  |
| 71-80 |  | - |  |  |  | - |  |  |  | - |  |  |  | - |  |  |  | - |  |  |  | - |  |  |
| 81- |  | -0.36 | (-1.65 , 0.93) |  |  | -0.55 | (-1.92 , 0.82) |  |  | 0.32 | (-1.04 , 1.68) |  |  | -0.66 | (-2.01 , 0.68) |  |  | -0.81 | (-2.09 , 0.47) |  |  | 0.31 | (-1.05 , 1.67) |  |

+ p<0.1, *p<0.05, ** p<0.01, ***p<0.001, Higher score indicates unfavorable stress-response. All betas were adjusted by age, type of job, job class, employment status, type of schedule, company size, company industry, job control, supervisors support and coworker’s support. Lack of vigor was derived by reversing the score of vigor for harmonization with other stress-response scales, which higher score indicates unfavorable stress-response

**Specific comment #13**

**Line 188: suggested wording: “perceived” in perceived lack of vigor” …**

**Line 193: suggested wording: “reported” in reported to be more vigorous at work …**

**Line 197: suggested wording: “reported” in reported significantly higher …**

(Our response for this comment)

We agree with all these suggestions, thus we revised the part as you suggested.

**Specific comment #14**

**Page 15**

**Line 204: When discussing differences between man and women perform a subgroup evaluation focusing on gender distribution in leading/non-leading positions.**

(Our response for this comment)

We appreciate this important suggestion. Recently, a study found that the mortality rate was different between non-leading and leading positions, and thus this stratified analysis needed to be conducted. The main findings were not affected considerably, compared with the previous one, However, these results were not covered sufficiently in past studies, therefore we added these results as table 4.

(Materials and methods section, page 11, line 168-171)

*Finally, stratified analyses by gender, age-category (i.e. less than 30, 30-59 and 60 or older) and job-position (regular and manager) were also performed. In these models, interaction terms between age-category/job-position and overtime working hours were used to explore possible effect modification by gender, age and job-position.*

(Results and discussions section, page 18, line 240-246)

*Table 3 and 4 showed stratified analysis results by age-category and job-class. Compared to middle-aged male workers, older workers showed relatively higher stress responses, especially fatigue, anxiety and depression. It may suggest that older workers constitute a more vulnerable population for overtime working than middle-aged workers. Due to the small number of working women in higher job positions, the interaction by job-class was not clear. It may be interesting that the highest beta coefficient in point estimates was observed for fatigue among women in administrative position (beta=1.16 [0.68. 1.63]).*

(Added tables)

Table 4: Association between overtime work hours and stress responses; results of multiple linear regression stratified by gender and job-class

**Specific comment #15**

**Line 206: When taking about selection-bias arising from different health conditions consider that you lack profound variables on physical and mental health.**

(Our response for this comment)

We appreciate this important suggestion. We agree with this comment because our data did not include any physical health data. Therefore, we revised this part.

(Result section, page 17, line 226-228)

*Both among men and women there is no increase in effect above 80 hours overtime work/month. Indeed the data did not include any clinical data, however speculatively, there might be a healthy worker effect in this extreme group.*

**Specific comment #16**

**Page 16/17, table 2**

**In Characteristics: suggested wording: Overtime work hours/month**

(Our response for this comment)

We agree with this suggestion, thus we revised the phrase in all tables. We appreciate this.

**Specific comment #17**

**Page 18**

**Line 223: Take into account that overtime work not necessarily implies being under time pressure and working at high speed.**

(Our response for this comment)

We appreciate this comment. Indeed, a past study suggests that work pace needs to be separated from amount of hours in the assessment of quantitative job demands (Kristensen et al 2006). Thus, even among those with the same amount of overtime, it may be possible that work pace would be different. We added this point in the limitations of Discussion section.

(Results and discussions section, page 32, line 387-393)

*Furthermore, overtime working hours were assessed by self-report. Although a previous study showed that measuring overtime through self-report had high validity and that reproducibility was confirmed among Japanese workers (57), self-reported overtime working hours may be less accurate than objectively measured working hours. In addition, this study did not consider work pace of overtime. Even among workers with the same length of overtime working, a higher stress response may be observed among those who are under severe time-pressure or experience a higher working pace (58).*

**Specific comment #18**

**Line 230: Health perceptions differ according to age and cultural background and cannot be considered independently of a personal sense of vigor. Consider that European Working Time Directives limit daily working time to 11 hours and weekly working time to 48 hours.**

**(http://ec.europa.eu/social/main.jsp?catId=706&langId=en&intPageId=205).**

(Our response for this comment)

We agree with this comment. The regulation of working hours differ between European and Asian countries. This information with the web-page were added, as follows.

(Results and discussions section, page 25, line 267-271)

*Especially, a recent systematic review shows that long working hours are related to higher risk of depression in Asian countries, compared to European countries (34).* *Indeed, working time regulation differed between European and Asian countries (35). However, the present study showed results compatible with previous studies and reinforced the evidence that longer working hours could have harmful effects on workers’ mental health.*

**Specific comment #19**

**Page 19**

**Line 241: Consider that the lifespan perspective of work design and ageing at work includes modification of job demands in relation to age**

(Our response for this comment)

We appreciate this comment. In according to your previous comment, we added age-stratified analysis. In result, men with older age showed higher beta coefficients in analysis of covariance. That implies that they may be a vulnerable population for overtime work hours.

(Results and discussions section, page 26, line 279-284)

*First, workers with long working hours need a sufficient amount of time for recovery from work, and these workers may not have enough time for relaxing or refreshing themselves after finishing work. This may be one possible reason why older workers showed higher stress responses than workers in middle age among with 21-50 hours/month of overtime. Since older adults may need more recovery time, they may be more vulnerable for overtime working.*

**Specific comment #20**

**Page 21**

**Line: 280: Consider that effects of work-to-family spillover and from job demands that interfere with the family domain involve men and women.**

(Our response for this comment)

We appreciate this comment. Work-family spillover would be one possible reason for the gender-gap of the association between vigor and overtime. Especially in Japan, working women spend much more time for child rearing or household chores than working men. Hence, long working women would be more exhausted or less vigorous than men.

This is very important point, therefore, we revised the Discussion part, as follows.

(Results and discussions section, page 28-29, line 319-331)

*Specific reasons for this gender-gap are unknown. However, self-selection into jobs with long working hours among men could be speculated. As discussed above, workers with high vigor may aspire to work longer hours. However, that may be difficult for women due to household responsibilities or other familial roles. Hence, in comparison to vigorous men, vigorous women may have relatively shorter length of working hours, which may be one possible cause of this gender-gap. The difference in work-family spillover between working men and women could be suggested as another possible explanatory factor. According to the Japanese Survey on Time use and Leisure activities in 2016, among couples with preschool children, the average time for child rearing or household chores were 84 min/week and 370 min/week for men and women, respectively (46). It is reported that work family negative spillover is more explicit in women, compared with men, and is related with psychological distress (47). Speculatively, women with long working hours may tend to suffer more from negative work-family spillover, compared to men.*
