# Supplementary material for: Association of overtime work hours with various stress responses in 59,021 Japanese workers: Retrospective cross-sectional study
Source: PLoS One. 2020 Mar 3;15(3):e0229506. doi: 10.1371/journal.pone.0229506 (PMC7053771; doi:10.1371/journal.pone.0229506)
Supplement: Attachment — Submitted filename: [Plos One, R2] Response to reviwers.docx [file pone.0229506.s004.docx]

Reviewer #1

I have gone through the revision of he manuscript entitled Association of overtime work hours with various stress responses in 59,021 Japanese workers: retrospective cross-sectional study. I think the authors were very responsive to the reviewer comments. In my view, the manuscript in its current form has the potential to make a meaningful contribution to the literature on overtime. Below I outline a couple of minor issues that ideally should be addressed to make the manuscript more reader-friendly.

**Comment #1**

On page 8 and 9 you state that you combined the categories 70-80 hours and 81+ hours to one category. To me it seems like this decision is not reflected in all the analyses presented. I suppose this statement occurs erroneously. Please align the statements in the methods section with the focal analyses.

**Our response for this comment**

We apologize this confusing notation. We used eight categories for overtime, i.e.“20 hours or less”, “21-30 hours”, “31-40 hours”, “41-50 hours”, “51-60 hours”, “61-70 hours”, “71-80 hours” and “81 hours or more”. The category of “81+” was generated after consolidating all categories from “81-90 hours“ to “140+ hours”. We revised the manuscript to clarify this point.

(Method section, page 8-9, line 122-127)

*This study assessed the length of participants’ monthly length of overtime working hours. Self-reported monthly overtime data was collected in increments of every 10 hours from “20 hours or less” to “141 hours or more”. Due to the smaller number of participants who engaged in 81 hours or more overtime we consolidated them into one category, and thus the present study set the categorization of overtime as the following 8 categories, “20 hours or less”, “21-30 hours”, “31-40 hours”, “41-50 hours”, “51-60 hours”, “61-70 hours”, “71-80 hours” and “81 hours or more”.*

**Comment #2&3**

I suggest you introduce the focal stress responses in the theory section. At a minimum, you should give readers (unfamiliar with the specific literature on overtime) an idea which facets of experienced stress you study why combining these variables makes sense. In response to one of my comments to added a discussion on work engagement, vigor etc. I suggest stating upfront which facets of individual stress responses your study is aimed at.

On a related note, in response to my comment in the last review, you added a section to argue for the differences between vigor as a facet of work engagement and vigor as a facet affect in terms of the POMS. According to this section both conceptualizations are different because they have been conceptualized by different authors or in different domains. In my view, such a statement is unfortunate for two reasons: First, it does not clarify what the actual differences are. Second, it is not a strong argument for distinguishing the two conceptualizations of vigor. I think, Shirom has worked out the conceptual overlap and distinctiveness between energy-related constructs in several chapters and papers. Just one idea: The vigor facet of work engagement is explicitly work-related. The vigor facet of the POMS is more generic. I don’t think it is necessary to call for future research on the work engagement vigor facet. However, I urge you to be precise regarding the definition and the conceptualization of the stress responses. Please state clearly upfront (in the theory section) what defines fatigue, vigor, depressive mood etc. and why specifically these stress responses do make sense as a bundle. I would explain at the beginning what vigor and fatigue have in common and to what extent they are different. Please note that this is a conceptual rather than an empirical question. You might find the literature on human energy as a unifying framework helpful (Quinn, Spreitzer, & Lam, 2012).

**Our response for these comments**

(Since these comments related with each other, we would like to respond together.)

First of all, we appreciate this thoughtful comment. As you pointed, theoretical consideration would be needed to interpret the findings in this paper. With regards to difference between vigor and fatigue, we thought the correlation between these two factors is informative for readers, so we added that information in results section. Furthermore we added some explanation in discussion section. These are from our thought that we would like this paper to put more focus on epidemiological contribution.

(Discussion part, Page 30-31, Line 341-352)

*Vigor among workers have attracted research attention in positive occupational health psychology for instance in studies of work engagement (50,51). Work engagement has been defined as “a positive, fulfilling, work-related state of mind that is characterized by vigor, dedication and absorption”(52). Vigor in work-engagement assessments has included concepts of “motivation” or “psychological resilience”, whereas the items for assessment of vigor in BJSQ have been based on POMS. According to Shirom, the conceptualization of vigor in both work-engagement and BJSQ is not exactly the same (53). In our study “lack of vigor” was measured from the POMS. This negative concept is related on a deeper theoretical level to fatigue. In fact, moderate correlations between “lack of vigor” and “fatigue” were observed (men: r=-0.328, women: r=-0.358). However, the distinction between these interrelated concepts remains unclear theoretically. Exploring the association between overtime working hours and vigor in work engagement is an important research question in future studies*

**Comment #4**

Please be more specific in the methods sections which stress response has been derived from the POMS and which has been derived from the other scales.

**Our response for this comment**

We appreciate this comment. We added detailed explanation how stress-response scales in the BJSQ were chosen into six scales.

(Method section, page 9, line 130-140)

*Stress responses were assessed using the Brief Job Stress Questionnaire (BJSQ) following the recommended protocol of the Stress Check Program (25–27). The BJSQ was originally designed to measure both psychological and somatic, both positive and negative stress responses among workers in any workplace with minimum number of items (28). Six stress-response scales can be measured by 29 questionnaire items in the BJSQ, and each items were developed by referring some already standardized/authorized questionnaires. In detail, “vigor”, “fatigue”, and “irritability” , consisting of 3 items each, were from the Profile Of Mood States (POMS). “Depression”, consisting of 6 items, was from the Center for epidemiologic Studies for Depression Scale (CES-D). “Anxiety”, consisting of 3 items, was from the State-Trail Anxiety Inventory (STAI). “Somatic stress responses”, consisting of 11 items, was from Screener for the Somatoform Disorders and the Subjective Wellbeing Inventory (SUBI)(28).*

**Comment #5**

I appreciate the clarification regarding the analyses. I think the analyses treating overtime as a continuous variable is most relevant. Consider making this analysis more focal. My impression is that – in the current version of the manuscript – you emphasize the regression models applying the overtime categories as dummy variables. For instance, in Table 2 you examine over and over again, whether 61 hours of overtime is different from less than 20 hours and whether 41 hours are different from less than 20 hours. I think the linear analysis and the dummy analyses test different hypotheses: Linear change vs. change after crossing a threshold of 21 hours. In my view, there are two ways to deal with this issue: First, make the threshold analyses less focal to the manuscript. Second, provide a rationale for conducting these competing views. Again, this would be very interesting and relevant from a theoretical perspective – although theoretical considerations don’t seem to be your primary aim with this manuscript. At a minimum, I would explain the rationale of the two competing views and the two sets of analyses more clearly in the paper – probably mentioning this aspect in the theory section already.

**Our response for this comment**

We appreciate this valuable comment. We agree with adding explanation why we use two statistical models simultaneously. Thus, we added explanations in the manuscript.

(Method section page 11-12, line 160-167)

*Multiple linear regression analysis was used to examine the associations of stress responses with overtime. In each model, we used a standardized score of each stress response (such as “lack of vigor” or “irritability”, etc.) as a dependent variable, “length of overtime work hours” as an independent variable, other individual characteristics (such as “age” and “job position”, etc.) as covariates. First, we performed linear trend tests by treating “length of overtime work hours” as a continuous variable to check the linear association between each stress response and overtime. Then, to seek possible thresholds of overtime, “length of overtime work hours” were treated as a dummy variable by setting “less than 20hours” group as a reference category.*

**Comment #6**

There is something like “lineally” in the manuscript. I am not sure whether this expression actually exists in English. Probably, “linearly” would be a better fit if you mean to refer to linear trajectories.

**Our response for this comment**

We apologize this mistake, thus we revised the part.

(Abstract section, page 3, line 45-46)

*Length of overtime was linearly associated with various stress responses, except for “lack of vigor”.*
